# Exploring the mechanistic link between *SF3B1* mutation and ring sideroblast formation in myelodysplastic syndrome

**DOI:** 10.1038/s41598-022-18921-2

**Published:** 2022-08-26

**Authors:** Tetsuro Ochi, Tohru Fujiwara, Koya Ono, Chie Suzuki, Maika Nikaido, Daichi Inoue, Hiroki Kato, Koichi Onodera, Satoshi Ichikawa, Noriko Fukuhara, Yasushi Onishi, Hisayuki Yokoyama, Yukio Nakamura, Hideo Harigae

**Affiliations:** 1 Department of Hematology, Tohoku University Graduate School of Medicine, 1–1 Seiryo-machi, Aoba-ku, Sendai, 980-8574 Japan; 2 Laboratory Diagnostics, Tohoku University Hospital, Sendai, Japan; 3 Department of Hematology-Oncology, Institute of Biomedical Research and Innovation, Foundation for Biomedical Research and Innovation at Kobe, Kobe, Japan; 4 Cell Engineering Division, RIKEN BioResource Research Center, Tsukuba, Ibaraki Japan

**Keywords:** Myelodysplastic syndrome, RNA splicing

## Abstract

Acquired sideroblastic anemia, characterized by bone marrow ring sideroblasts (RS), is predominantly associated with myelodysplastic syndrome (MDS). Although somatic mutations in *splicing factor 3b subunit 1* (*SF3B1*), which is involved in the RNA splicing machinery, are frequently found in MDS-RS, the detailed mechanism contributing to RS formation is unknown. To explore the mechanism, we established human umbilical cord blood-derived erythroid progenitor-2 (HUDEP-2) cells stably expressing SF3B1^K700E^. SF3B1^K700E^ expressing cells showed higher proportion of RS than the control cells along with erythroid differentiation, indicating the direct contribution of mutant SF3B1 expression in erythroblasts to RS formation. In SF3B1^K700E^ expressing cells, *ABCB7* and *ALAS2,* known causative genes for congenital sideroblastic anemia, were downregulated. Additionally, mis-splicing of *ABCB7* was observed in SF3B1^K700E^ expressing cells. *ABCB7*-knockdown HUDEP-2 cells revealed an increased frequency of RS formation along with erythroid differentiation, demonstrating the direct molecular link between ABCB7 defects and RS formation. ALAS2 protein levels were obviously decreased in *ABCB7*-knockdown cells, indicating decreased ALAS2 translation owing to impaired Fe–S cluster export by ABCB7 defects. Finally, RNA-seq analysis of MDS clinical samples demonstrated decreased expression of *ABCB7* by the *SF3B1* mutation. Our findings contribute to the elucidation of the complex mechanisms of RS formation in MDS-RS.

## Introduction

Sideroblastic anemia comprises a group of congenital and acquired disorders that share the characteristic presence of bone marrow (BM) ring sideroblasts (RS), which contain excess mitochondrial deposits of iron^1–4^. Congenital sideroblastic anemia (CSA) is a rare condition that constitutes a diverse class of inherited disorders. Based on the pathophysiology of mitochondrial iron-heme metabolism, CSA-causative genes can be categorized into the following three subtypes: heme biosynthesis-associated genes, including *5-aminolevulinate synthase* (*ALAS2*), *solute carrier family 25 member 38* (*SLC25A38*), and *ferrochelatase* (*FECH*); Fe–S cluster biosynthesis-associated genes, including *ATP binding cassette subfamily B member 7* (*ABCB7*), *heat shock protein family A member 9* (*HSPA9*) and *glutaredoxin 5* (*GLRX5*); and genes associated with mitochondrial protein synthesis^1–7^. The most prevalent form of CSA is X-linked sideroblastic anemia (XLSA), which is attributed to mutations in the X-linked erythroid-specific *ALAS2* gene, which encodes the first rate-limiting enzyme in heme biosynthesis^1,2^. ALAS2 expression is mainly regulated by GATA-binding protein 1 (GATA-1), a master regulator of erythropoiesis^8^.

Acquired sideroblastic anemia without obvious etiologies, such as lead toxicity or copper deficiency, frequently accompanies myelodysplastic syndrome (MDS), which are bone marrow failures characterized by dysplasia and high frequencies of leukemic transformation^9^. Although RS can be observed irrespective of MDS subtype, MDS with more than 15% RS in BM falls into a distinct category called MDS with RS (MDS-RS), often accompanied by somatic mutations in splicing factor 3b, subunit 1 (*SF3B1*)^10^. While the prevalence of *SF3B1* mutation is 20–28% in the entire MDS population^11^, mutation frequencies in MDS with RS (MDS-RS) are higher, with 80% and 40% for MDS-RS with single lineage dysplasia (MDS-RS-SLD) and multilineage dysplasia (MDS-RS-MLD), respectively^12^. Thus, the revised World Health Organization classification proposed that if a demonstrable *SF3B1* gene mutation is identified, MDS-RS can be diagnosed if RS comprise as few as 5% of nucleated erythroid cells; otherwise, at least 15% RS is still required for the definite diagnosis of MDS-RS^13^.

SF3B1 is the largest component of the U2 small nuclear ribonucleoprotein (snRNP), which plays an important role in recognizing the branchpoint sequence (BPS), polypyrimidine tract (PyT), and 3′ splice site (3′ SS) in RNA splicing^14^. SF3B1 prevents aberrant splicing by strengthening the connection between the spliceosome and premature mRNA through interaction with p14 and U2 Small Nuclear RNA Auxiliary Factor 1 (U2AF1) and U2AF2 via the N-terminal HEAT domain (which consists of repeated alpha helices)^15^. Mutations in the HEAT domain can trigger aberrant splicing, especially the usage of alternative 3′ SS (A3SS), due to the recognition of alternative BPS caused by changes in the positional relationship between mRNA and spliceosome^16^. Among the various types of *SF3B1* mutations that largely exist in the HEAT domain, the p.K700E mutation in exon 15 is the most frequent^17^.

Despite the strong association between *SF3B1* mutations and RS emergence in MDS, the detailed molecular mechanisms by which *SF3B1* mutations contribute to RS formation remain elusive. The expression level of *ABCB7*, a CSA-causative gene, is lower in MDS-RS cases than in MDS non-RS cases^5,18^, probably due to induced abnormal splicing of *ABCB7* in *SF3B1* mutated *(SF3B1*^*MUT*^*-*) MDS cases^19^. However, little is known regarding the detailed molecular mechanism by which SF3B1 mutation contribute to RS formation.

The hematopoietic stem cell-specific *Sf3b1*^*K700E*^ knock-in mouse model exhibited anemia, while did not reproduce RS formation nor exhibited aberrant splicing of *Abcb7*, which is considered a key event in RS formation^20^. Although exhibition of RS formation in the *SF3B1*^*G742D*^-MDS-RS model cell has been reported^21^, the establishment of an MDS-RS model harboring *SF3B1*^*K700E*^ is desirable because MDS with the *SF3B1*^*K700E*^ mutation was reported to be different from MDS with other SF3B1 mutations in terms of splicing pattern and prognosis^22^. In addition, as the *SF3B1*^*G742D*^-MDS-RS model was derived from induced pluripotent stem cells (iPSCs) of an MDS-RS patient in which the additive chromosomal abnormality of t(4;12)(q31.3;q15) co-existed with the *SF3B1*^*G742D*^ mutation^21,23^, the possibility of the potential contribution of co-existing chromosomal abnormalities on RS formation might not be ruled out.

Recently, we succeeded in establishing culture conditions to induce RS using XLSA models^24,25^. Using this methodology for human umbilical cord blood-derived erythroid progenitor-2 (HUDEP-2) cells^26^, we aimed to reveal the detailed molecular mechanisms of RS formation induced by SF3B1^K700E^.

## Results

### Differentiated HUDEP-2 cells stably expressing SF3B1^K700E^ exhibited RS formation

To examine the direct link between the expression of SF3B1^K700E^ and RS formation, we established HUDEP-2 cells stably expressing SF3B1^K700E^, which were subsequently induced to undergo erythroid differentiation by co-culturation with OP-9 cells (see Supplementary Fig. S1a-b online). Control vector-transduced HUDEP-2 cells and HUDEP-2 cells stably expressing SF3B1^WT^ were used as controls. Expression of codon optimized *SF3B1*^*WT*^ or *SF3B1*^*K700E*^ were confirmed by RT-PCR and Sanger sequencing (see Supplementary Fig. S1c and S3 online).

**Figure 1:**
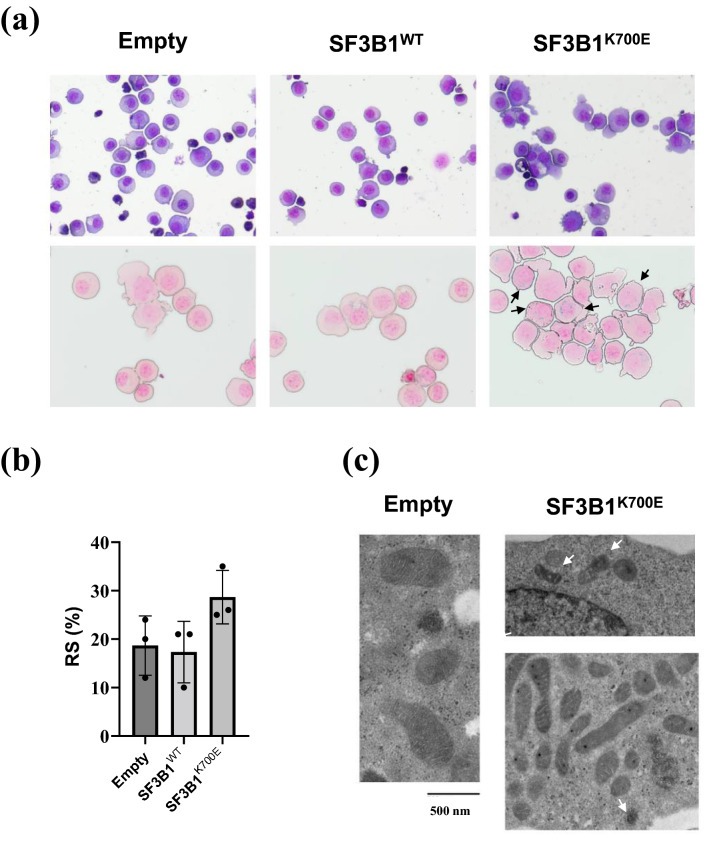
RS formation of HUDEP-2 cells stably expressing SF3B1^K700E^ after differentiation. (**a**) Representative micrograph of cytospin slides for differentiated HUDEP-2 cells stably expressing SF3B1^K700E^ or controls. The upper photographs show slides stained with May–Grünwald–Giemsa stain, and the lower ones represent those stained with Prussian blue. Ring sideroblasts are indicated by black arrows. (**b**) RS proportion of differentiated HUDEP-2 cells stably expressing SF3B1^K700E^ or controls are shown as mean ± SD and dot plots. (**c**) Representative electron micrographs of differentiated HUDEP-2 cells stably expressing SF3B1^K700E^ or controls. Electron dense deposits in mitochondria are indicated by white arrows.

Quantitative RT-PCR revealed specifical expression of codon optimized *SF3B1* in HUDEP-2 cells stably expressing SF3B1^WT^ or SF3B1^K700E^ and global expression of internal *SF3B1* in each cell line, although the expression of level of internal *SF3B1* was significantly higher in HUDEP-2 cells stably expressing SF3B1^K700E^ when compared to controls (see Supplementary Fig. S4 online). The relatively low expression level of codon optimized *SF3B1*^*K700E*^ compared to that of codon optimized *SF3B1*^*WT*^ might imply the survival inferiority of HUDEP-2 cells expressing SF3B1^K700E^ at a high level. The higher level of internal *SF3B1* in HUDEP-2 stably expressing SF3B1^K700E^ than in controls could be explained as compensatory mechanism for inhibiting mis-splicing caused by abnormal SF3B1 in HUDEP-2 cells stably expressing SF3B1^K700E^. May–Grünwald–Giemsa staining confirmed erythroid differentiation into polychromatic and orthochromatic erythroblasts (Fig. 1a). The proportion of RS was higher in HUDEP-2 cells stably expressing SF3B1^K700E^ than in controls (Fig. 1a, b). Electron microscopic observation of HUDEP-2 cells stably expressing SF3B1^K700E^ revealed mitochondria containing electron-dense deposits, indicating abnormal iron accumulation (Fig. 1c).

### Gene expression analysis for HUDEP-2 cells stably expressing SF3B1^K700E^

**Figure 2:**
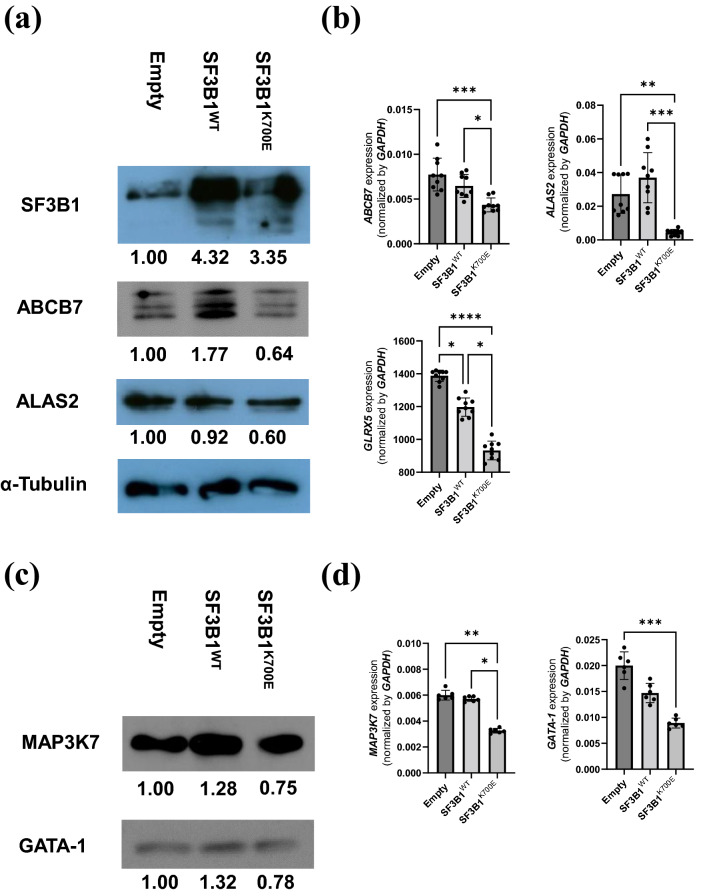
Gene expression analysis of HUDEP-2 cells stably expressing SF3B1^K700E^. (**a**) Western blot analysis for SF3B1, ABCB7, and ALAS2. Relative expression level of each gene in HUDEP-2 cells stably expressing SF3B1^WT^ or SF3B1^K700E^ in comparison to control vector-transduced HUDEP-2 cells are described under each picture. α-Tubulin was used as a loading control. The image of each protein was cropped from the different films. The original films are presented in Supplementary Fig. S6–9 online. (**b**) Expression levels of *ABCB7*, *MAP3K7* and *GLRX5* were measured by quantitative RT-PCR (results shown as mean ± SD and dot plots); * p < 0.05, ** p < 0.01, *** p < 0.001, **** p < 0.0001. (**c**) Western blot analysis for MAP3K7 and GATA-1. Relative expression level of each gene in HUDEP-2 cells stably expressing SF3B1^WT^ or SF3B1^K700E^ in comparison to control vector-transduced HUDEP-2 cells is described under each picture. α-Tubulin was used as a loading control (**a**). The image of each protein was cropped from the different films. The original films are presented in Supplementary Fig. S10–11 online. (**d**) Expression levels of *MAP3K7* and *GATA-1* were measured by quantitative RT-PCR (results shown as mean ± SD and dot plots); * p < 0.05, ** p < 0.01, *** p < 0.001.

To detect candidate genes contributing to RS formation in HUDEP-2 cells stably expressing SF3B1^K700E^, we performed RNA-seq analysis based on HUDEP-2 cells stably expressing SF3B1^K700E^ and controls. Comprehensive gene expression analysis with RNA-seq did not show apparent changes in HUDEP-2 cells stably expressing SF3B1^K700E^ in the expression levels of CSA-causative genes, except *ALAS2* (see **S**upplementary Table S1 online). Western blotting revealed downregulation of ALAS2 and ABCB7 in HUDEP-2 cells stably expressing SF3B1^K700E^ (Fig. 2a). Quantitative RT-PCR demonstrated significantly decreased expression levels of *ABCB7*, *ALAS2* and *GLRX5* (Fig. 2b). These results suggested the contribution of some dysregulated CSA-causative genes to RS formation in MDS-RS.

Moreover, comprehensive gene expression analysis with RNA-seq revealed downregulation of GATA-1 target genes, including *ALAS2*, solute carrier family 4 member 1 (*SLC4A1*)^27^, ankyrin-1 (*ANK1*)^28^, and aminolevulinate dehydrogenase (*ALAD*)^29^ in HUDEP-2 cells stably expressing SF3B1^K700E^ (see Supplementary Table S3 online). Decreased expression levels of *mitogen-activated protein kinase 7* (*MAP3K7*) and *GATA-1* in HUDEP-2 cells stably expressing SF3B1^K700E^ were described by quantitative RT-PCR and western blotting (Fig. 2c, d). In contrast, quantitative RT-PCR for differentiated HUDEP-2 cells did not show dysregulation of *ALAS2*, *MAP3K7*, *ABCB7*, *GATA-1* and *GLRX5* by SF3B1^K700E^ expression (see Supplementary Fig. S5 online). Combined with the suggested role of MAP3K7 to phosphorylate p38MAPK associated with regulating GATA-1 function^30,31^, SF3B1^K700E^ expression could result in compromised GATA-1 protein expression presumably mediated by downregulation of *MAP3K7*.

### Alternative splicing analysis for HUDEP-2 cells stably expressing SF3B1^K700E^

**Figure 3:**
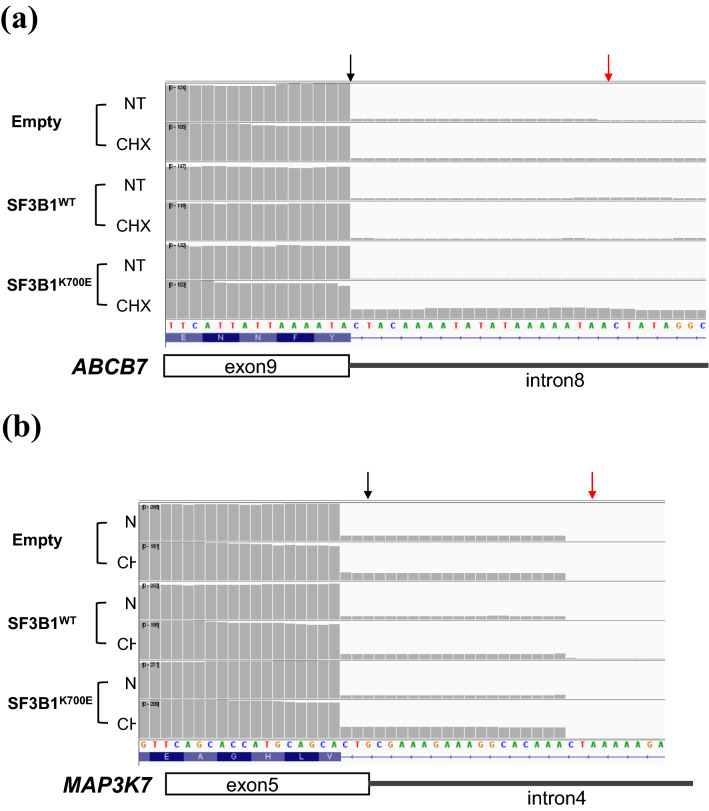
Detection of A3SS events in *ABCB7* and *MAP3K7* with HUDEP-2 cells stably expressing SF3B1^K700E^ with or without CHX treatment. (a-b) Read-coverage visualized by IGV around canonical 3′ SS of *ABCB7* exon 9 (**a**) and canonical 3′ SS of *MAP3K7* exon 5 (**b)**. Black and red arrow indicate canonical and aberrant 3′ SS, respectively. Empty, SF3B1^WT^ and SF3B1^K700E^ indicate HUDEP-2 cells transduced with control vector, HUDEP-2 cells stably expressing SF3B1^WT^ and HUDEP-2 cells stably expressing SF3B1^K700E^, respectively. NT and CHX indicate the non-treated samples and the samples treated with CHX, respectively.

We subsequently explored the contribution of aberrant splicing induced by SF3B1^K700E^ to the differential gene expression. Read-coverage visualization with Integrative Genomics Viewer (IGV)^32^ revealed an increased number of reads mapped to intron 8 of *ABCB7* and intron 4 of *MAP3K7* in HUDEP-2 cells stably expressing SF3B1^K700E^ after cycloheximide (CHX) treatment (Fig. 3). These results indicated the existence of SF3B1^K700E^-induced A3SS events as previously reported^19,33^, leading to the production of splice variants targeted by nonsense-mediated decay (NMD).

Moreover, we performed comprehensive alternative splicing (AS) analysis using Mixture of Isoforms (MISO)^34^. MISO analysis did not detect significant differences in the number of significant AS events between HUDEP-2 cells stably expressing SF3B1^WT^ and SF3B1^K700E^ when compared with control vector-transduced HUDEP-2 cells (see Supplementary Fig. S12a, Tables S3-4 online). Significant AS events in HUDEP-2 cells stably expressing SF3B1^K700E^ were not detected in *ALAS2* and *GLRX5*, suggesting that these genes would not be downregulated through aberrant splicing induced by SF3B1^K700E^ (see Supplementary Tables S4 online). Although the number of A3SS events detected in HUDEP-2 cells stably expressing SF3B1^WT^ or SF3B1^K700E^ was equivalent, most of the events did not overlap. HUDEP-2 cells stably expressing SF3B1^K700E^ exhibited the significant A3SS event in *ribonuclease/angiogenin inhibitor 1* (*RNH1*) (see Supplementary Fig. S12b online)^33^. Although aberrantly spliced isoforms were detected even in control vector-transduced HUDEP-2 cells by RT-PCR, the sashimi plot described by MISO revealed a significant increase in A3SS usage between exons 2 and 3 of *RNH1* in HUDEP-2 cells stably expressing SF3B1^K700E^ (see Supplementary Fig. S13a-b online). Sanger sequencing of RT-PCR products revealed that A3SS caused the addition of 68 bases within the 5′ untranslated region (UTR) of *RNH1* (see Supplementary Fig. S13c online). However, we could not detect any difference in the expression level of RNH1 either at mRNA or at protein level between HUDEP-2 cells stably expressing SF3B1^K700E^ and controls (see Supplementary Fig. S13d-e online).

Thus, we demonstrated that ABCB7 could be mainly downregulated via increased A3SS usage between exons 8 and 9 induced by SF3B1^K700E^. Moreover, downregulation of ALAS2 at the mRNA level was suggested to be associated with decreased GATA-1 function, probably caused by downregulation of *MAP3K7* because of increased A3SS usage between exons 4 and 5.

### Confirmation of mis-splicing–mediated downregulation of ABCB7 and MAP3K7 in K562 cells

**Figure 4:**
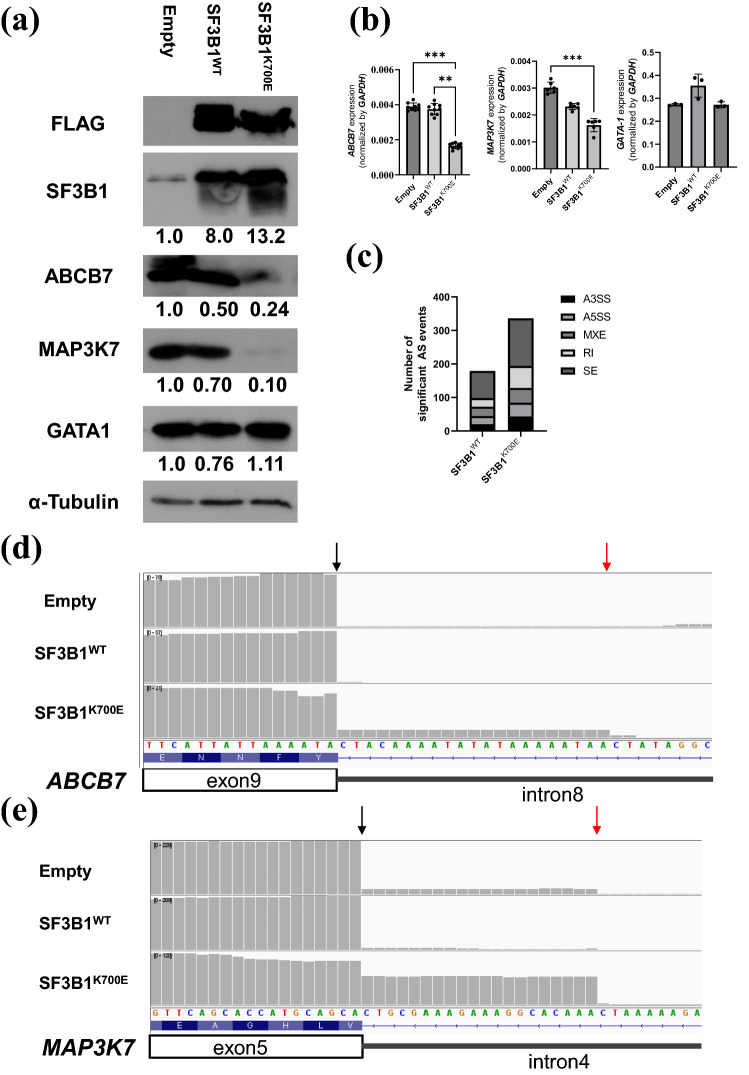
Gene expression analysis of K562 cells overexpressing SF3B1^K700E^. (**a**) Western blot analysis for FLAG, SF3B1, ABCB7, MAP3K7, ALAS2, and GATA-1. Relative expression level of each gene in K562 cells expressing SF3B1^WT^ or SF3B1^K700E^ in comparison to control vector-transduced K562 cells are described under each picture. α-Tubulin was used as a loading control. The image of each protein was cropped from the different fields of the film. The original film is presented in Supplementary Fig. S17–21 online. (**b**) Expression levels of *ABCB7, MAP3K7* and *GATA-1* by quantitative RT-PCR (results shown as mean ± SD and dot plots); ** p < 0.01, *** p < 0.001. (**c**) Comprehensive AS analysis with MISO. The graph shows the number of significant AS events detected in K562 cells stably expressing SF3B1^WT^ or SF3B1^K700E^ when compared with control vector-transduced K562 cells. (**d**, **e**) Read-coverage visualized by IGV around canonical 3′ SS of *ABCB7* exon 9 (**d**) and canonical 3′ SS of *MAP3K7* exon 5 (**e**). Black and red arrow indicate canonical and aberrant 3′ SS, respectively. Empty, SF3B1^WT^ and SF3B1^K700E^ indicate K562 cells transduced with control vector, K562 cells overexpressing SF3B1^WT^ and K562 cells overexpressing SF3B1^K700E^, respectively.

Although downregulation of *MAP3K7* and *ABCB7* possibly caused by mis-splicing was detected in HUDEP-2 cells stably expressing SF3B1^K700E^, the incidence of A3SS events in *MAP3K7* and *ABCB7* was so low that the detection of A3SS events required NMD inhibition with CHX. To reinforce the evidence for mis-splicing–mediated downregulation of *ABCB7* and *MAP3K7* induced by SF3B1^K700E^, K562 cells overexpressing SF3B1^K700E^ or SF3B1^WT^ were generated by electroporation (see Supplementary Fig. S16 online), as confirmed using western blotting (Fig. 4a). Repeated electroporation resulted in stronger expression of SF3B1 in K562 cells expressing SF3B1^WT^ or SF3B1^K700E^ than in HUDEP-2 cells stably expressing SF3B1^WT^ or SF3B1^K700E^. Similar to the experiment with HUDEP-2 cells (Fig. 2), quantitative RT-PCR and western blotting confirmed significantly decreased expression levels of ABCB7 and MAP3K7 both at mRNA and protein levels in K562 cells expressing SF3B1^K700E^ compared to those in control vector-transduced K562 cells or K562 cells expressing SF3B1^WT^ (Fig. 4a, b). The number of significant AS events detected by MISO analysis was higher in K562 cells expressing SF3B1^K700E^ than in those expressing SF3B1^WT^ when compared with control vector-transduced K562 cells (Fig. 4c). Lists of significant AS events can be found in Supplementary Table S5-6. Read-coverage visualization with IGV detected A3SS events between exons 4 and 5 of *MAP3K7* and between exons 8 and 9 of *ABCB7* in K562 cells expressing SF3B1^K700E^ even without CHX treatment (Fig. 4d, e).

### ABCB7-knockdown in HUDEP-2 cells lead to RS formation

**Figure 5:**
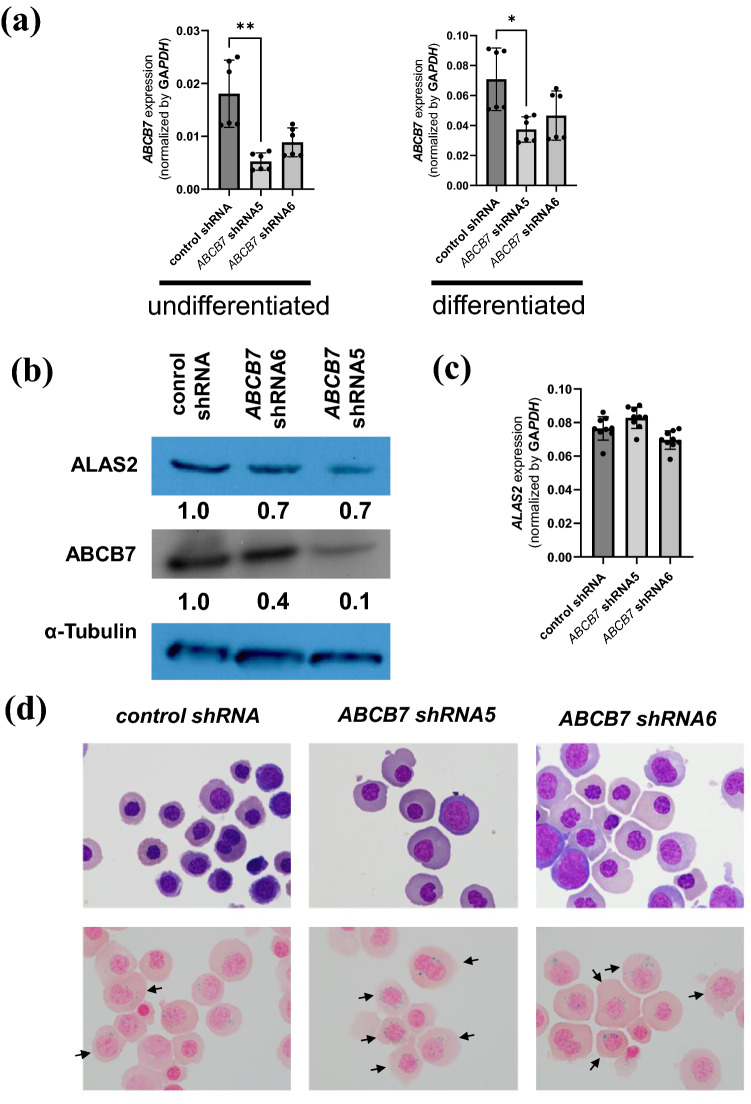
Analysis of *ABCB7*-knockdown HUDEP-2 cells. (**a**) Expression levels of *ABCB7* by quantitative RT-PCR (results shown as mean ± SD and dot plots) in undifferentiated and differentiated *ABCB7*-knockdown HUDEP-2 cells; * p < 0.05, ** p < 0.01. (**b**) Western blot analysis for ALAS2, ABCB7 and α-Tubulin in undifferentiated *ABCB7*-knockdown HUDEP-2 cells. Relative expression levels of ALAS2 and ABCB7 in *ABCB7*-knockdown HUDEP-2 cells in comparison to control shRNA-transduced HUDEP-2 cells described under the picture. α-Tubulin was used as a loading control. The image of each protein was cropped from the different fields of the film. The original film is presented in Supplementary Fig. S23-24 online. (**c**) Expression levels of *ALAS2* by quantitative RT-PCR (results shown as mean ± SD and dot plots) in undifferentiated *ABCB7*-knockdown HUDEP-2 cells. (**d**) Representative micrograph of cytospin slides for differentiated *ABCB7*-knockdown HUDEP-2 cells. The upper photographs show slides stained with May–Grünwald–Giemsa stain, and the lower ones represent those stained with Prussian blue. Ring sideroblasts are indicated by black arrows. Control, shRNA5, and shRNA6 represent HUDEP-2 cells transduced with control shRNA, HUDEP-2 cells transduced with *ABCB7* shRNA clone 5, and HUDEP-2 cells transduced with *ABCB7* shRNA clone 6, respectively.

**Figure 6:**
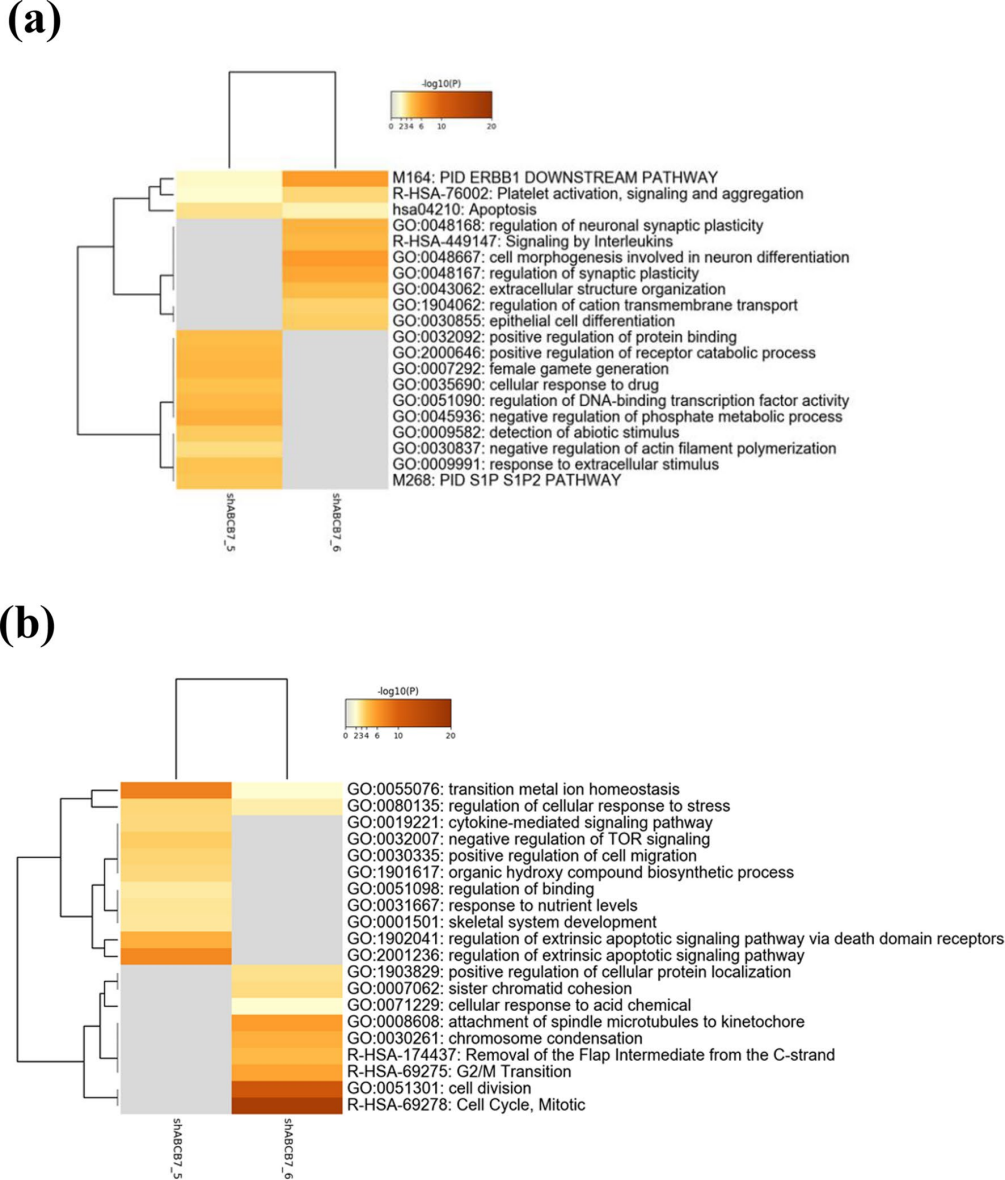
Gene ontology enrichment analysis of dysregulated genes in *ABCB7*-knockdown HUDEP-2 cells. Enrichment heatmap of genes (**a**) upregulated and (**b**) downregulated by *ABCB7*-knockdown. *ABCB7*_shRNA5 and *ABCB7*_shRNA6 indicate HUDEP-2 cells transduced with *ABCB7* shRNA clone 5 and HUDEP-2 cells transduced with *ABCB7* shRNA clone 6, respectively.

Although *ABCB7* is known to be one of the genes responsible for CSA^1^, the direct contribution of *ABCB7* defects to RS formation has not been demonstrated. Thus, we conducted shRNA–mediated *ABCB7*-knockdown in HUDEP-2 cells, which were subsequently induced to undergo erythroid differentiation (see Supplementary Fig. S22 online). *ABCB7*-knockdown was confirmed both at mRNA and protein levels in undifferentiated HUDEP-2 cells, especially using shRNA clone 5 (Fig. 5a, b). Decreased expression level of *ABCB7* in differentiated *ABCB7*-knockdown HUDEP-2 was confirmed by quantitative RT-PCR, unless western blotting could not be performed (Fig. 5a). Although there was no significant change in *ALAS2* mRNA expression levels (Fig. 5c), ALAS2 protein levels were noticeably decreased by *ABCB7*-knockdown, indicating decreased *ALAS2* translation (Fig. 5b). The progression of erythroid differentiation was also morphologically confirmed by May–Grünwald–Giemsa staining (Fig. 5d, upper panel). Prussian blue staining revealed an increased frequency of RS formation in *ABCB7*-knockdown HUDEP-2 cells compared with that in control shRNA-transduced HUDEP-2 cells (Fig. 5d, lower panel). Expression profiling analysis revealed that 39 and 20 genes were commonly upregulated and downregulated by more than 1.5-fold, respectively, in *ABCB7*-knockdown HUDEP-2 cells compared with control cells (see Supplementary Table S7 online). Gene ontology (GO) enrichment analysis by Metascape revealed significant enrichment of genes involved in iron metabolism and apoptosis among the commonly upregulated and downregulated genes, respectively (Fig. 6).

Collectively, we demonstrated a direct link between *ABCB7* defects and RS formation, which might be associated with decreased ALAS2 translation.

### Analysis of gene expression and AS using clinical samples

**Figure 7:**
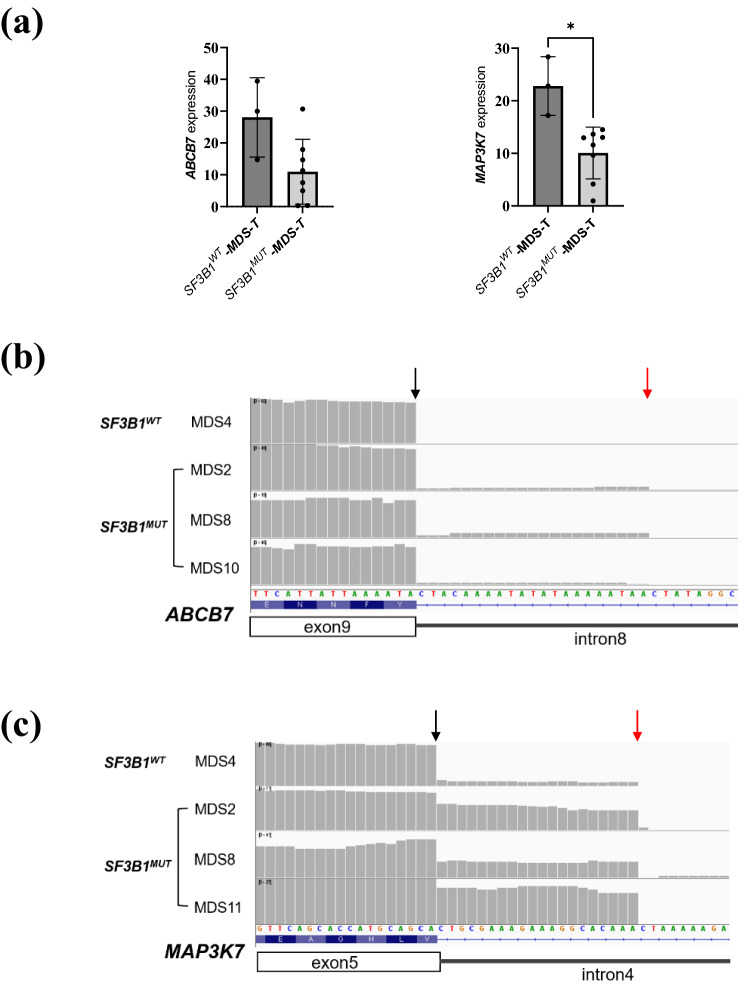
Analysis of MDS clinical samples. (**a**) RNA-seq analysis of *ABCB7* and *MAP3K7* expression levels in *SF3B1*^*WT*^- or *SF3B1*^*MUT*^-MDS patients diagnosed at Tohoku University Hospital; * p < 0.05. (**b**, **c**) Read-coverage visualized with IGV around canonical 3′ SS of *ABCB7* exon 9 (**b)** and of *MAP3K7* exon 5 (**c**) in representative MDS patients diagnosed at Tohoku University Hospital. *SF3B1*^*WT*^- or *SF3B1*^*MUT*^-MDS-T refers to the RNA-seq data of MDS patients diagnosed at Tohoku University Hospital.

To confirm the contribution of downregulation of *ABCB7* and *MAP3K7* to RS formation in *SF3B1*^*MUT*^-MDS, we analyzed the RNA-seq data of clinical samples derived from MDS patients diagnosed at Tohoku University Hospital. Our cohort included eight *SF3B1*^*MUT*^-patients (two MDS-SLD, five MDS-RS-SLD, and one MDS-EB1) and three *SF3B1*^*WT*^-MDS patients (one MDS-RS-SLD, one MDS-RS-MLD, and one MDS-MLD) (see Supplementary Table S8 and Fig. S25a online). As shown in Fig. 7a, the expression levels of *MAP3K7* and *ABCB7* were lower in *SF3B1*^*MUT*^-MDS patients than in *SF3B1*^*WT*^-MDS patients, although not significantly lower for *ABCB7*. On the other hand, there was no difference in the expression levels of *ABCB7* and *MAP3K7* between MDS-RS and non MDS-RS patients (see Supplementary Fig. S26 online), indicating a greater contribution of *SF3B1* mutation status, rather than RS existence, to the regulation of *ABCB7* and *MAP3K7* expression. RNA-seq read-coverage of *ABCB7* and *MAP3K7* visualized by IGV revealed previously reported AS events in some *SF3B1*^*MUT*^-MDS patients (Fig. 7b, c), although the incidence of A3SS between exons 8 and 9 of *ABCB7* seemed very low. AS events in *ABCB7* and *MAP3K7* accompanied with lower expression levels of *ABCB7* and *MAP3K7* in *SF3B1*^*MUT*^-MDS patients indicate downregulation of *ABCB7* and *MAP3K7* due to degradation of mis-spliced transcripts by NMD. Additionally, the A3SS event between exons 2 and 3 of *RNH1* detected in HUDEP-2 cells expressing SF3B1^K700E^ was confirmed in both *SF3B1*^*WT*^- and *SF3B1*^*MUT*^-MDS patients, whereas the PSI of this event was relatively high in *SF3B1*^*MUT*^-MDS patients (See Supplementary Fig. S27 online).

Lastly, we analyzed RNA-seq dataset GSE114922 obtained from GEO RNA-seq Experiments Interactive Navigator (GREIN)^35^ for validating downregulation of *ABCB7* and *MAP3K7* in a large MDS cohort. GSE114922 contains RNA-seq data of BM CD34 positive cells derived from 54 *SF3B1*^*WT*^*-* and 28 *SF3B1*^*MUT*^-MDS patients^36^. Similar to our findings (Fig. 7), the expression levels of *ABCB7* and *MAP3K7* were significantly lower in *SF3B1*^*MUT*^-MDS patients than in *SF3B1*^*WT*^-MDS patients, which was also confirmed for the cohort excluding CMML (Chronic myelomonocytic leukemia) and RAEB (Refractory anemia with excess blasts) (see Supplementary Fig. S28 online). Furthermore, we aimed to find the unique role of SF3B1^K700E^ as compared with SF3B1^non-K700E^. The expression levels of *ABCB7* and *MAP3K7* were compared among *SF3B1*^*WT*^-, *SF3B1*^*K700E*^-, and *SF3B1*^*non-K700E*^-MDS patients in both whole cohort and subgroups (Refractory anemia [RA] and RA with ring sideroblasts [RARS]) (see Supplementary Fig. S29–30 online). However, we could not identify any differential impact on the expression levels of these genes between *SF3B1*^*K700E*^- and *SF3B1*^*non-K700E*^-MDS patients. Then, we performed differentially expressed genes (DEGs) analysis with the web tool, iDEP^37^, for overviewing the expression profile of *SF3B1*^*WT*^-, *SF3B1*^*K700E*^- and *SF3B1*^*non-K700E*^-MDS patients. When compared to *SF3B1*^*WT*^-MDS patients, the number of DEGs identified in *SF3B1*^*K700E*^- and *SF3B1*^*non-K700E*^ MDS was 1130 (324 genes were upregulated and 806 genes were downregulated) and 27 (2 genes were upregulated and 25 genes were downregulated), respectively (see Supplementary Fig. S31 online). Gene ontology (GO) enrichment analysis by Metascape^38^ revealed significant enrichment of the genes involved in oxygen transport and erythroid differentiation among the upregulated genes and those involved in the productivities or responsivities of cytokines among the downregulated genes in *SF3B1*^*K700E*^-MDS patients (see Supplementary Fig. S32 online).

These data imply that SF3B1^K700E^ may exert more comprehensive impact on gene expression than SF3B1^non-K700E^.

## Discussion

In this study, we successfully established HUDEP-2 cells expressing SF3B1^K700E^ as an MDS-RS model (Fig. 1). This model exhibited downregulation of *ABCB7*, *ALAS2*, and *GLRX5* (Fig. 2), all of which are known CSA-causative genes, indicating a cross-link between acquired and congenital SA.

*ABCB7*, located at Xp13.3, encodes a mitochondrial transporter of the Fe–S cluster and is responsible for XLSA with ataxia (XLSA/A)^1^. The mis-splicing–associated downregulation of *ABCB7* detected in our MDS-RS model (Figs. 2a, b, 3a) is closely associated with MDS-RS^5,18^. As *Abcb7*-knockout mice do not survive^39^, RS formation by *ABCB7* defects in erythroid cells has never been reproduced. Here, we firstly established *ABCB7*-deficient RS model by *ABCB7*-knockdown in HUDEP-2 cells (Fig. 5). This model showed decreased expression levels of ALAS2 at the protein level, but not at the mRNA level (Fig. 5b, c), indicating impaired ALAS2 translation by *ABCB7*-knockdown. ALAS2 translation is inhibited when iron regulatory protein 1 (IRP1) binds iron-responsive element (IRE) located in the 5′ UTR of *ALAS2* mRNA, but is promoted when IRP1 combined with Fe–S cluster is converted to aconitase lacking the ability to bind IRE^40^; hence, we speculated that a decrease in cytosolic Fe–S cluster owing to downregulation of *ABCB7* could contribute to RS formation by inhibiting ALAS2 translation via IRP1 activation. Further analyses are required to assess changes in aconitase activity and IRP/IRE interactions.

The molecular link between ALAS2 defects and RS formation has already been previously demonstrated^24,25^. Comprehensive AS analysis for HUDEP-2 cells stably expressing SF3B1^K700E^ did not detect any AS events in ALAS2 (see Supplementary Table S4 online), indicating unlikeliness of mis-splicing–mediated downregulation of ALAS2. However, we observed downregulation of GATA-1 target genes, such as *ALAS2*^8^, *SLC4A1*^26^, *ANK1*^27^, and *ALAD*^29^, and confirmed decreased GATA-1 protein levels (Fig. 2c). Thus, we focused on *MAP3K7* as the cause of GATA-1 dysregulation in our MDS-RS model, because *MAP3K7*, which is activated by TGF-β, is known to phosphorylate p38MAPK, regulating GATA-1 function both by phosphorylating GATA-1 and by promoting ubiquitination and proteasomal degradation of GATA-1 via MAPKAKP2/HSP27^30,31^. Our MDS-RS model exhibited mis-splicing–associated downregulation of *MAP3K7* (Figs. 2c, d, 3b), which was confirmed in K562 cells overexpressing SF3B1^K700E^ (Fig. 4a, b and e) and *SF3B1*^*MUT*^-MDS patients (Fig. 7a and c)*.* Downregulation of *MAP3K7* mediated by mis-splicing has been reported to decrease the expression level of GATA-1 in SF3B1^K700E^-mutated K562 cells^33^. Additionally, our MDS-RS model showed increased PSI of the A3SS event within 5′ UTR of *RNH1* (see Supplementary Fig. S13 online), which was also confirmed in *SF3B1*^*MUT*^-MDS patients (see Supplementary Fig. S27 online). RNH1 could be broadly detected in various human tissues including enucleated erythroid cells lacking RNase^41^. RNH1 has been reported not only as the indispensable factor for survival and development of mice due to its protective role of global RNA from RNase but also as the translational regulator for the specific genes including GATA-1^42,43^. We hypothesized that impaired RNH1 translation associated with increased A3SS usage within 5′ UTR induced by mutant SF3B1 might contribute to the downregulation of ALAS2 at the transcription level by enhancing impaired GATA-1 translation in *SF3B1*^*MUT*^-MDS. Although we failed to demonstrate decreased RNH1 translation followed by impaired GATA-1 translation in HUDEP-2 stably expressing SF3B1^K700E^, this could be confirmed by a novel approach like polysome profiling or western blotting for each fraction of the cells. Taken together, we speculate that *ALAS2* downregulation mediated by GATA-1 dysfunction may play a role in RS formation induced by SF3B1^K700E^ expression.

Our MDS-RS model exhibited *GLRX5* downregulation (Fig. 2b). Comprehensive AS analysis did not indicate mis-splicing–mediated downregulation of *GLRX5* (see Supplementary Table S4 online). Although the mechanism by which downregulation of *GLRX5* was induced by SF3B1^K700E^ is unknown, it might be mediated by the downregulation of *ABCB7* because *ABCB7*-knockdown downregulates GLRX5^44^. The role of GLRX5 as a supplier of Fe–S cluster to IRP1^45^ suggests that downregulation of *GLRX5* could also inhibit ALAS2 translation via IRP1 activation, such as downregulation of *ABCB7*.

The inner mitochondrial membrane protein TMEM14C which functions in the final steps of heme synthesis as the transporter of protoporphyrinogen IX into mitochondrial matrix is highly expressed in the erythroid cells at the terminal differentiation steps^46^. *TMEM14C* expression is not under the control of IRP-IRE system^46^, while transcriptionally regulated by GATA-1^8^. According to iPSC-derived *SF3B1*^*G742D*^-MDS-RS model, coordinated mis-splicing *TMEM14C* and *ABCB7* contributed to RS formation^21^. In our models, increased frequency of the A3SS event was observed in K562 overexpressing SF3B1^K700E^ and some *SF3B1*^*MUT*^-MDS patients, but not in HUDEP-2 stably expressing SF3B1^K700E^ (see Supplementary Fig. S33–34 online). We speculated that lower expression level of SF3B1^K700E^ compared to endogenous SF3B1 (see Supplementary Fig. S4 online) might make it difficult to detect obvious *TMEM14C* mis-splicing. Further analysis based on more physiological model, such as CRISPR/Cas9-mediated SF3B1 mutation in HUDEP-2 cells, would be preferred to demonstrate the detailed mechanism of RS formation by SF3B1 mutation.

**Figure 8:**
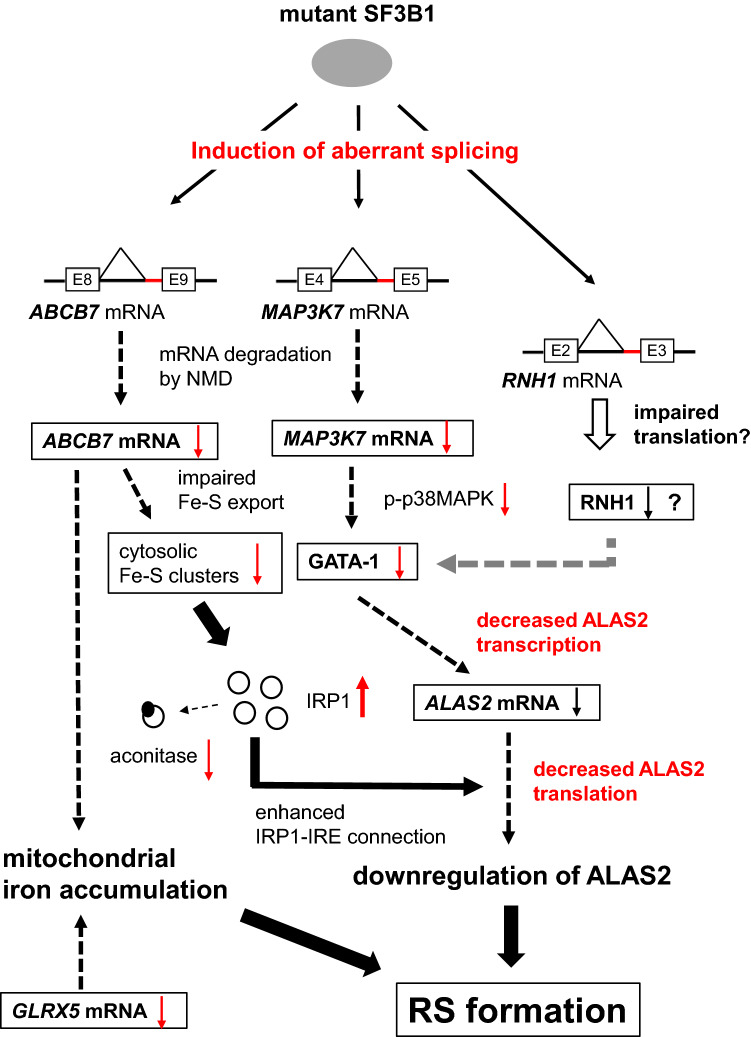
Proposed mechanism for RS formation in *SF3B1*^*MUT*^-MDS. A3SS usage in *ABCB7*, *MAP3K7*, and *RNH1* is promoted by the spliceosome containing the mutant SF3B1. Targeting of increased mis-spliced *ABCB7* mRNA by NMD contributes to the downregulation of ABCB7, resulting in reduced translation of ALAS2 through an enhanced IRP1-IRE system induced by reduced cytosolic Fe–S cluster. Increased mis-spliced *MAP3K7* mRNA, also targeted by NMD, contributes to the downregulation of MAP3K7, causing deterioration of GATA-1 function through the reduction in phosphorylated p38MAPK, as previously described^31^. Mis-splicing in the 5′ UTR of *RNH1* might impair translation of RNH1, leading to downregulation of RNH1, which was reported to downregulate GATA-1 by inhibition of translation^42^. Thus, transcription of ALAS2 is reduced owing to decreased GATA-1 function induced by downregulation of MAP3K7, and perhaps RNH1. In conclusion, downregulation of ALAS2 at both the transcriptional and translational levels because of mutant SF3B1-induced mis-splicing in *ABCB7*, *MAP3K7*, and possibly *RNH1* could be considered the underlying mechanism of RS formation in *SF3B1*^*MUT*^-MDS. Moreover, downregulation of *ABCB7* and *GLRX5* also promoted RS formation by accelerating mitochondrial iron accumulation through decreased Fe–S cluster export from the mitochondria and decreased production of Fe–S cluster, resulting in decreased use of mitochondrial free iron. p-p38MAPK indicates phosphorylated p38MAPK.

In conclusion, our findings have delineated a complex mechanism for RS formation, including dysregulation of *ABCB7*, *ALAS2*, *GATA-1*, and *MAP3K7* expression (Fig. 8). Additionally, according to the observations on *ABCB7*-knockdown HUDEP-2 cells, downregulation of ABCB7 could impair ALAS2 translation presumably through via IRP1 activation. These results imply a complicated mechanism for RS formation in *SF3B1*^*MUT*^-MDS. Further characterization of the established MDS-RS model will aid in clarifying its molecular etiology and establishing novel therapeutic strategies.

## Methods

### Ethical statement

Informed consent was obtained from all patients. The protocol of this study was approved by the Institutional Review Board of Tohoku University Graduate School of Medicine and was based on the ethical principles for medical research involving human subjects of the Helsinki Declaration.

### Cell culture

The culture protocol for human erythroleukemia cell line K562 cells (ATCC CCL-243™, Manassas, VA), human embryonic kidney 293 T (HEK293T) cells, and HEK293T derived packaging cell line Plat-GP cells has been described previously^26,47^.

HUDEP-2 cells^26^ were maintained in StemSpan serum-free expansion medium (STEMCELL Technologies, Vancouver, BC, Canada), supplemented with 50 ng/mL stem cell factor (SCF; PEPROTECH, Rocky Hill, NJ), 3 IU/mL erythropoietin (EPO; Kyowa Hakko Kirin, Tokyo, Japan), 1 μg/mL doxycycline (DOX; Sigma-Aldrich, St. Louis, MO), and 1 μM dexamethasone (DEX; Sigma-Aldrich). The mouse mesenchymal stromal cell line OP9 cells (ATCC) was maintained in α-minimum essential medium (Thermo Fisher Scientific, Waltham, MA) supplemented with 20% (v/v) fetal bovine serum (FBS; Biological Industries USA, Cromwell, CT) and 1% (v/v) penicillin/streptomycin (Sigma-Aldrich). To induce erythroid differentiation, HUDEP-2 cells were seeded onto OP9 cells for 6–7 days, as previously described^25^.

### Codon-optimized SF3B1^WT^ or SF3B1^K700E^ overexpression

As human *SF3B1* sequences are toxic to *Escherichia coli*^48^, we used codon-optimized *SF3B1*^*WT*^ and *SF3B1*^*K700E*^ expression vectors (Addgene Plasmid #82576 and #82577, respectively)^49^, encoded in pcDNA (Invitrogen, Carlsbad, CA). Each coding sequence was cloned into the retroviral vector pBABE-puro (Addgene Plasmid #1764)^50^.

For transient overexpression of *SF3B1*^*WT*^ or *SF3B1*^*K700E*^, each pcDNA-based expression vector (10 μg) was transfected into K562 cells using the Amaxa Cell Line Nucleofector II (Lonza, Cologne, Germany) with the program T-016^27,47^. For retroviral overexpression of *SF3B1*^*WT*^ or *SF3B1*^*K700E*^, the pBABE-puro-based expression vector and VSV-G (Addgene plasmid #12259) were co-transfected into Plat-GP packaging cell lines (Cell Biolabs, San Diego, CA) using FuGene HD (Promega, Madison, WI). Seventy-two hours after transfection, viral supernatant was used for infection. After spin infection of HUDEP-2 cells at 1300 × g for 2 h, 1 μg/mL puromycin (Sigma-Aldrich) was added to the medium to select the transduced cells.

Primers used for amplification or detection of codon-optimized *SF3B1*^*WT*^ or *SF3B1*^*K700E*^ expression are shown online in Supplementary Table S9.

### Detection of SF3B1 mutation

Genomic DNA was extracted from whole BM lysates of MDS patients or cell lysates of *SF3B1* mutant cell lines using a DNeasy Blood & Tissue kit (Qiagen N.V., Hulsterweg, Netherlands). Mutations within *SF3B1* exons 14 to 16, where most *SF3B1* mutations exist, were screened using high-resolution melting analysis as previously reported^51^, followed by confirmation of the mutations with Sanger sequences when screening tests were positive. The primer sequences used for Sanger sequencing are listed online in Supplementary Table S9.

### shRNA–mediated ABCB7-knockdown in HUDEP-2 cells

Lentiviral-based knockdown of the human *ABCB7* gene was conducted with pGIPZ lentiviral shRNAmir (Clone ID: V3LHS_406787) (Open Biosystems, Huntsville, USA), as described previously^47^. The lentiviral vectors VSV-G and psPAX2 (Addgene plasmid #12260) were co-transfected into HEK293T cells; 72 h after transfection, the viral supernatant was used for infection, as in the retroviral overexpression protocol.

### Quantitative RT-PCR

Quantitative RT-PCR were conducted as described previously^27^. Primers used for quantitative RT-PCR are listed online in Supplementary Table S9.

### Expression profiling analysis

For RNA-seq analysis, total RNA was extracted from whole BM cells of MDS patients (see Supplementary Table S8 online) or cell pellets when analyzing cell lines using TRIzol reagent. For library preparation, the SMARTer Ultra Low RNA Kit (Illumina, San Diego, CA) and Illumina TruSeq stranded mRNA Library kit were used for clinical samples and the NEBNext Ultra II RNA Library Prep Kit for Illumina was used for cell lines. Libraries were sequenced on an Illumina NovaSeq6000 (Otogenetics, Norcross, GA, USA). Sequence data were mapped to the human reference genome, hg19/GRCh37, using HISAT2 (version 2.2.1) (http://daehwankimlab.github.io/hisat2/)^52^. Normalized expression level of each gene was calculated as transcripts per million using StringTie (version 2.1.7) (https://ccb.jhu.edu/software/stringtie/)^53^.

Microarray analysis was conducted using a Human Oligo chip 25 k (Toray, Tokyo, Japan), and subsequent GO enrichment analyses were performed using Metascape^38^. For global normalization, the background value was subtracted and subsequently adjusted to an average signal value of 25, and genes with > 100 were analyzed.

DEGs analysis for GSE114922 dataset was performed with web tool, iDEP.951 (http://bioinformatics.sdstate.edu/idep/)^37^ based on normalized count data obtained from GREIN. We selected DESeq2 method for DEGs identification setting false discovery rate cut off at 0.05 and a minimum fold change at 1.5.

### Alternative splicing analysis

The AS events of each sample were analyzed using MISO software (version 0.5.4) (https://miso.readthedocs.io/en/fastmiso/) with exon-centric analysis separately for A3SS, alternative 5′ splice site (A5SS), mutually exclusive exons (MXE), retained introns (RI) and skipping exons (SE)^34^. Human genome (hg19) alternative events v2.0 (https://miso.readthedocs.io/en/fastmiso/annotation.html), was used for MISO annotation. Among AS events extracted by the “compare miso” command, we considered the AS events passing all filtering criteria as significant, which in our study is as follows: (a) at least one inclusion read and one exclusion read, such that (b) the sum of inclusion and exclusion reads is at least 10, (c) the Δ PSI (percent spliced in) is ≥ 0.20, and (d) the Bayes factor is at least 10. AS events were expressed with visualized read-coverage using IGV^32^ or the Sashimi plot command in IGV or MISO.

### Cycloheximide treatment

As splice variant isoforms containing premature stop codons (PTCs) are targeted by NMD, which decreases the number of splice variant isoforms^54^, the less expressed splice variant isoforms are sometimes difficult to detect. Cycloheximide (Nacalai Tesque, Inc., Japan), an NMD inhibitor, was added to HUDEP-2 cells stably expressing SF3B1^WT^ or SF3B1^K700E^ at a final concentration of 100 ug/mL^19^.

### Production of cytospin slides and staining

Cytospin preparation and staining with May–Grünwald–Giemsa stain (Merck KgaA, Darmstadt, Germany) or Prussian blue (ScyTek Laboratories, Inc., West Logan, UT) were performed as described previously^25^. RS was defined as erythroblasts with ≥ five iron granules surrounding at least one-third of the nuclear lesion^55^.

### Electron microscopy

An electron microscope (H-7600; Hitachi) was used. The protocols for sample preparation have been described by Saito et al.^25^.

### Western blot analysis

Western blotting was conducted using whole-cell extracts, as described previously^25^. The expression level was quantified by densitometry with ImageJ^56^, relative to the expression level of the control. The primary antibodies used were as follows: α-Tubulin (CP06; EMD Millipore, Billerica, MA, USA), SF3B1 (27684-1-AP; Proteintech, Rosemont, USA), ALAS2 (ab184964; Abcam, Cambridge, UK), ABCB7 (LS-B13035, Lifespan BioSciences, Seattle, WA, USA), MAP3K7 (#5206, Cell Signaling Technology, Danvers, MA, USA), RNH1 (10345-1-AP, Proteintech, USA), FLAG (#14793, Cell Signaling Technology) and GATA-1 (#3535, Cell Signaling Technology).

### Statistical analysis

All statistical analyses were performed using GraphPad Prism 9 (GraphPad Software, San Diego, CA, USA, http://www.graphpad.com/). Nonparametric analysis was adopted because of the small sample size. The Mann–Whitney U test was used for comparing two groups and the Kruskal–Wallis test for comparing equal or more than three groups, followed by Dunn’s test. Statistical significance was set at p < 0.05.

## Supplementary Information

Supplementary Figures.

Supplementary Tables.

## Data Availability

Raw sequence data obtained by RNA-seq discussed in this study are available in the DDBJ Sequenced Read Archive (DRA) under accession numbers DRX315782 to DRX315799 and DRX337379 to DRX 337383. We obtained RNA-seq dataset GSE114922 deposited by Pellagatti et al.^36^ from GREIN (http://www.ilincs.org/apps/grein/)^34^. Microarray data of *ABCB7*-knockdown HUDEP-2 cells can be found in Supplementary Table S7 available with the online version of this article. The Lists of significant AS events detected in HUDEP-2 cells stably expressing SF3B1^WT^ or SF3B1^K700E^ when compared with control vector-transduced HUDEP-2 cells and those detected in K562 cells stably expressing SF3B1^WT^ or SF3B1^K700E^ when compared with control vector-transduced K562 cells can be found in Supplementary Table S3-6 available with the online version of this article.
