# Supplementary material for: Exploring the mechanistic link between SF3B1 mutation and ring sideroblast formation in myelodysplastic syndrome
Source: Sci Rep. 2022 Aug 26;12:14562. doi: 10.1038/s41598-022-18921-2 (PMC9418223; doi:10.1038/s41598-022-18921-2)
Supplement: Supplementary file 1 — Supplementary Figures. [file 41598_2022_18921_MOESM1_ESM.pdf]

Supplementary Figure S1

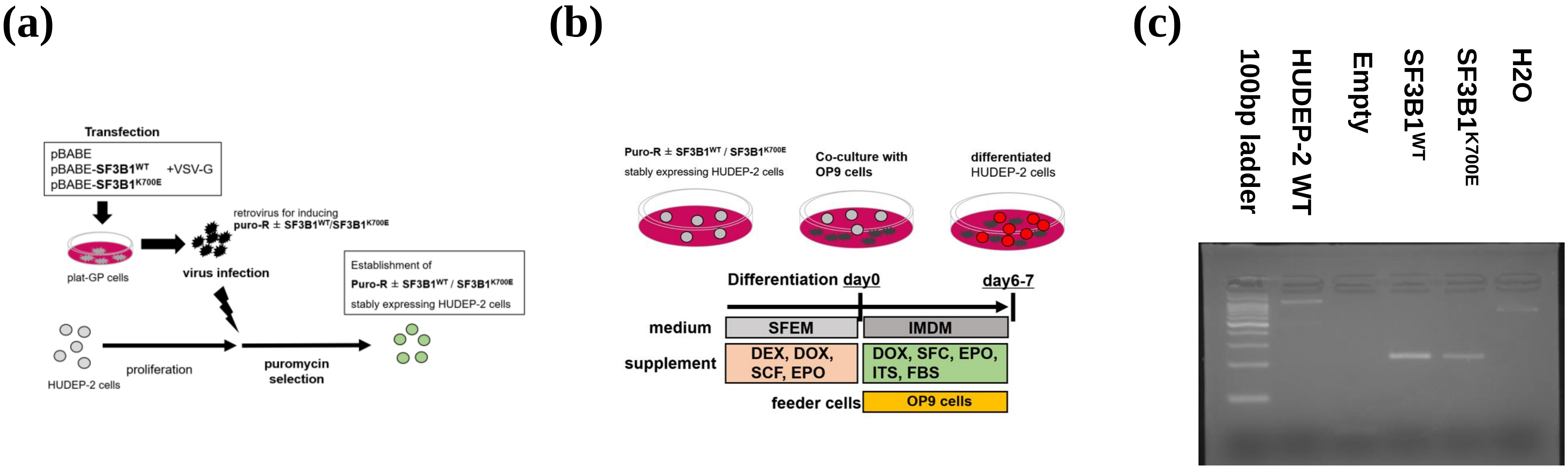

Supplementary Figure S1

Establishment and differentiation of HUDEP-2 cells stably expressing SF3B1^{K700E}.

(a) Schematic outline of the procedure for establishment of HUDEP-2 stably expressing SF3B1^{K700E}.

(b) Schematic outline of the procedure for differentiation induction of HUDEP-2 cells.

(c) Confirmation of codon-optimized *SF3B1* expression by RT-PCR. The original gel image is presented in **Supplementary Figure S2**.

Empty, SF3B1^{WT} and SF3B1^{K700E} indicate HUDEP-2 cells transduced with control vector, HUDEP-2 cells stably expressing SF3B1^{WT} and SF3B1^{K700E}, respectively.

Supplementary Figure S2

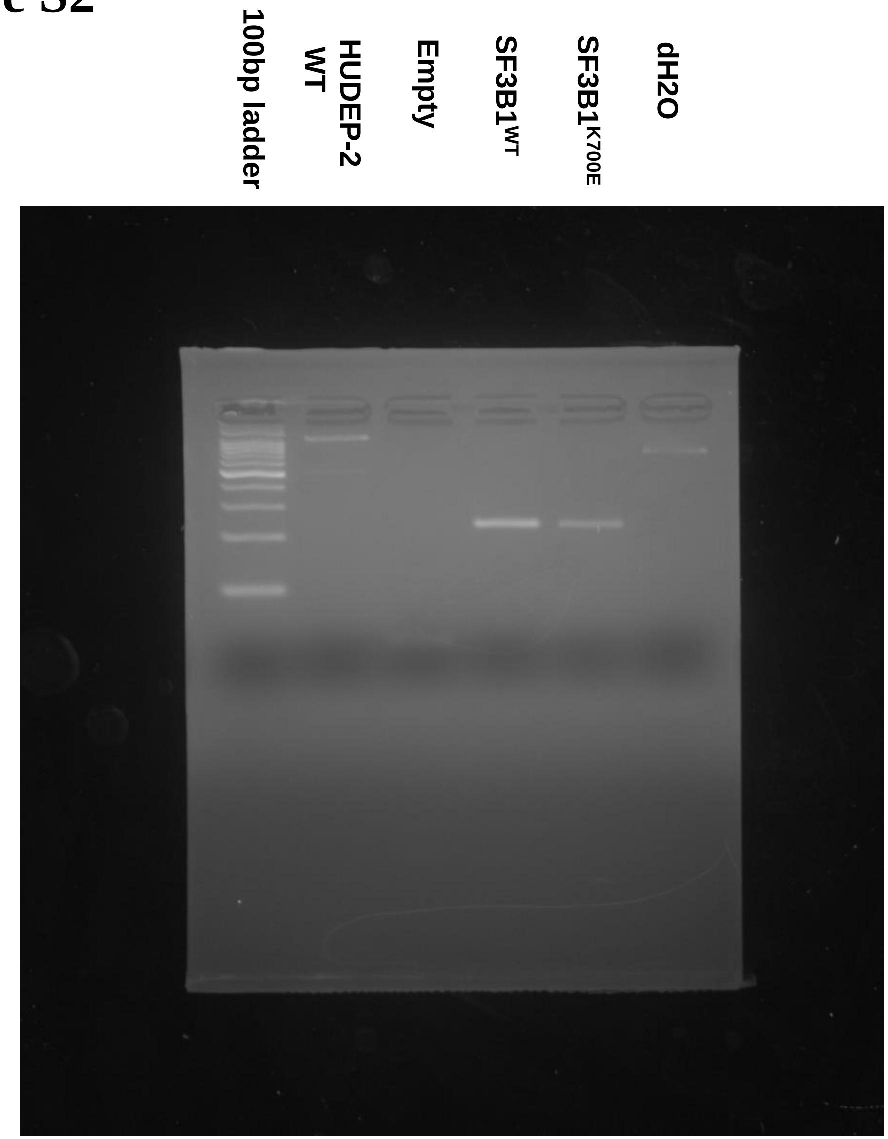

Supplementary Figure S2

Original gel image of Supplementary Fig. S1c.

Supplementary Figure S3

(a)

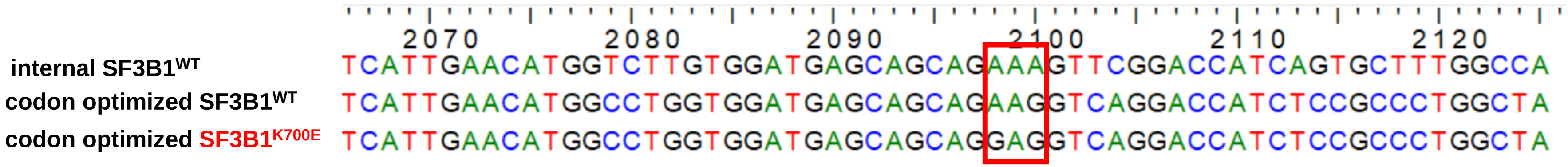

(b)

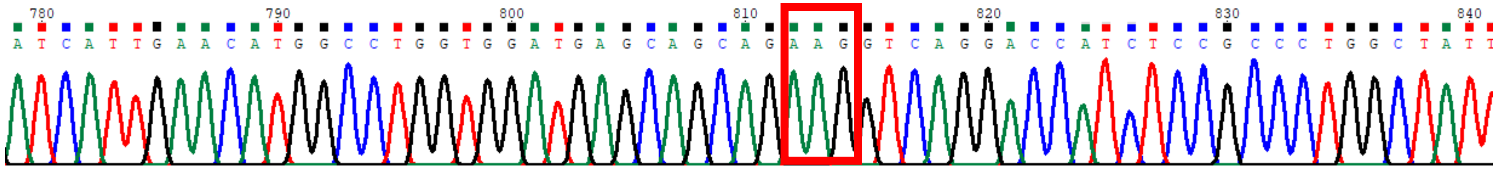

(c)

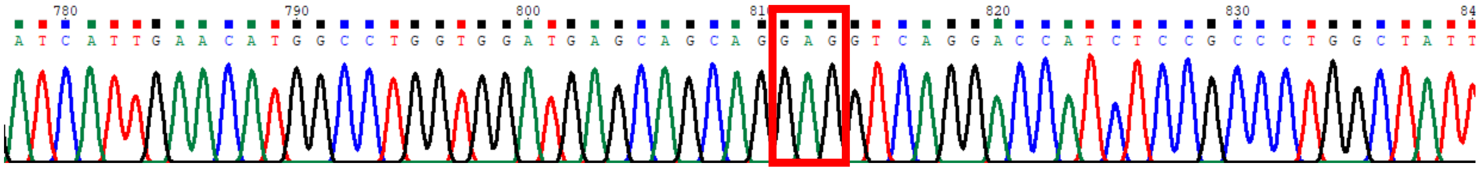

Supplementary Figure S3

Confirmation of codon optimized $SF3B1^{WT}$/$SF3B1^{K700E}$ expression in HUDEP-2 cells.

Amplified codon optimized SF3B1 sequences shown in Supplementary Fig. 1c were sequenced by Sanger method.

(a) Comparison of internal $SF3B1^{WT}$ and codon optimized $SF3B1^{WT}$/$SF3B1^{K700E}$ sequences.

(b) (c) Chromatogram of sequenced codon optimized $SF3B1^{WT}$ (b) and codon optimized $SF3B1^{K700E}$ (c).

c.2098-2100 (corresponding to p. 700) in each figure was highlighted by red boxes.

Supplementary Figure S4

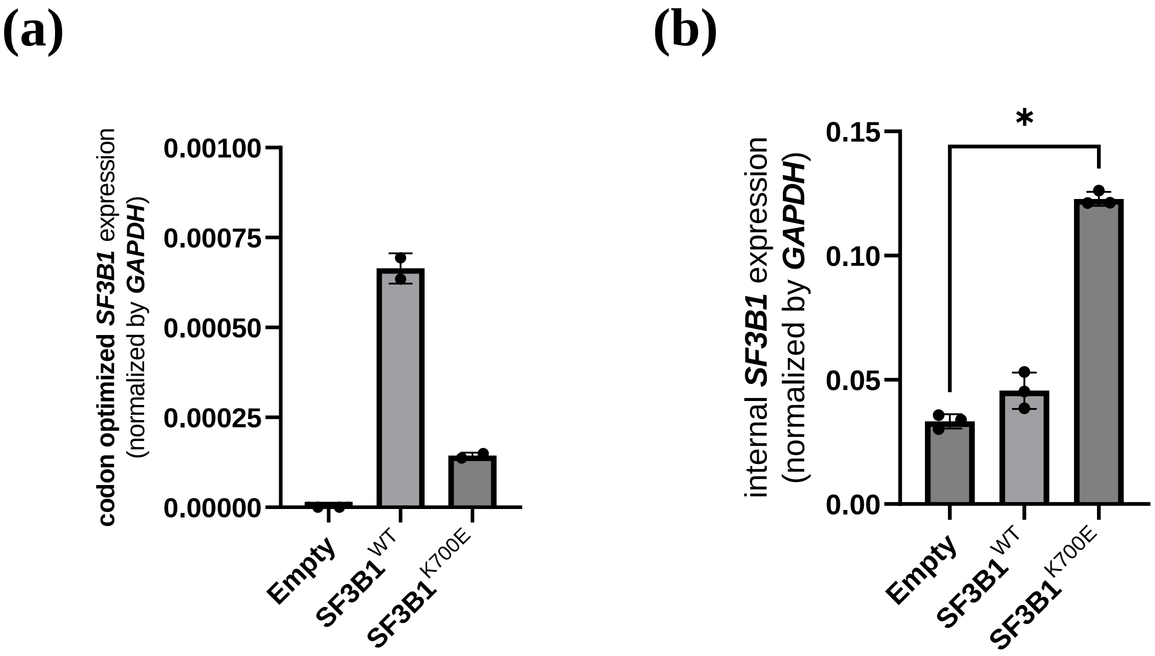

Supplementary Figure S4

Expression levels of internal SF3B1 and codon optimized SF3B1^{WT}/SF3B1^{K700E} in HUDEP-2 cells.

(a) (b) The expression levels of internal SF3B1 (a) and codon optimized SF3B1 (b) were quantified by RT-PCR. Empty, SF3B1^{WT} and SF3B1^{K700E} indicate HUDEP-2 cells transduced with control vector, HUDEP-2 cells stably expressing SF3B1^{WT} and SF3B1^{K700E}, respectively.

Supplementary Figure S5

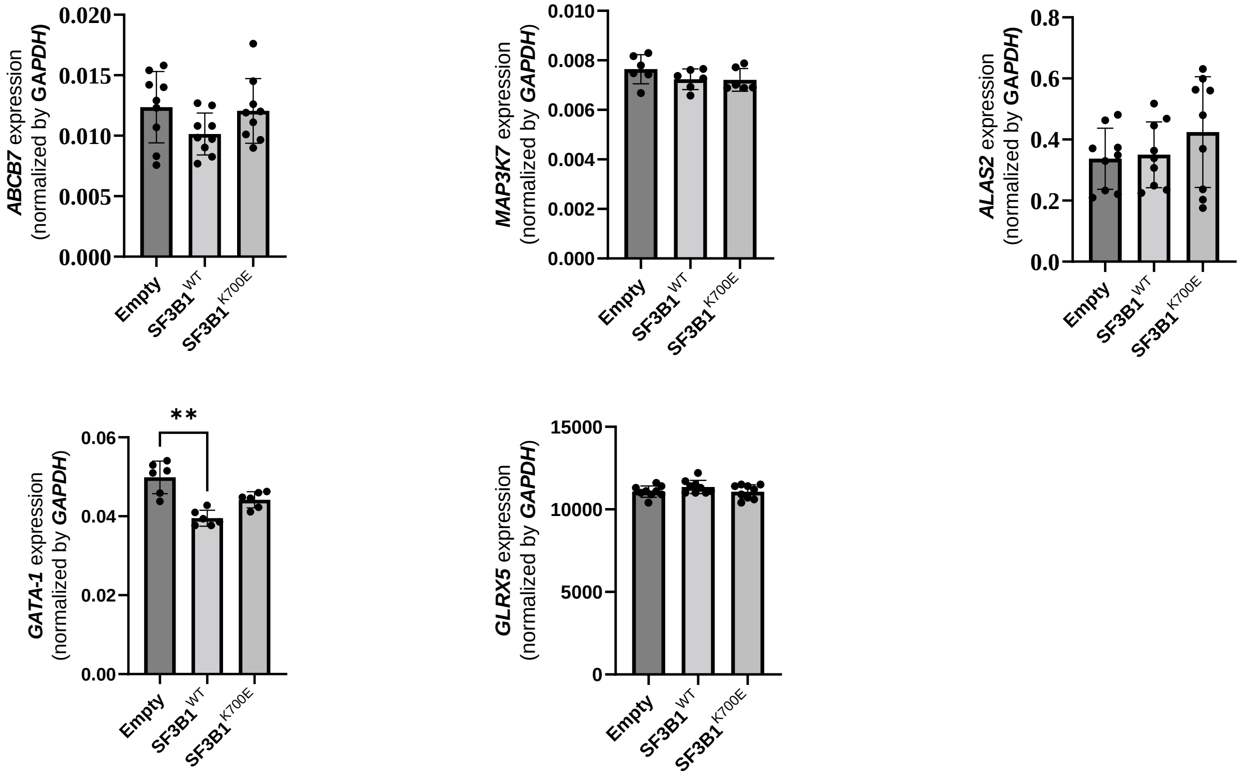

Supplementary Figure S5

Gene expression analysis of differentiated HUDEP-2 cells stably expressing SF3B1^{K700E} .

Expression levels of *ABCB7*, *GLRX5*, *ALAS2*, *MAP3K7* and GLRX5 were measured by quantitative RT-PCR (results shown as mean ± SD and dot plots). * $p < 0.05$.

Empty, SF3B1^{WT} and SF3B1^{K700E} indicate HUDEP-2 cells transduced with control vector, HUDEP-2 cells stably expressing SF3B1^{WT} and HUDEP-2 cells stably expressing SF3B1^{K700E}, respectively.

Supplementary Figure S6

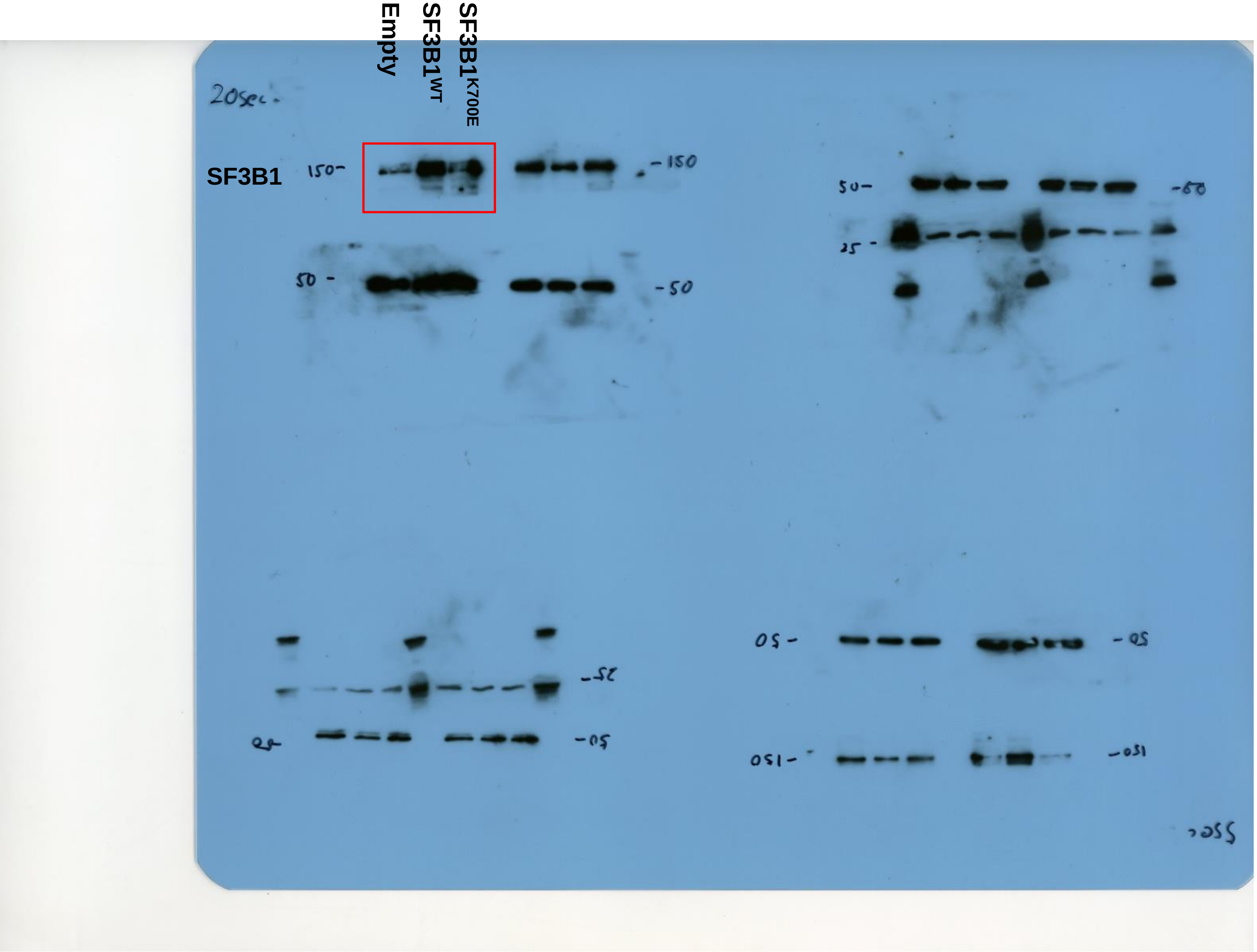

Supplementary Figure S6

Original blot image of SF3B1 in Fig. 2a.

Supplementary Figure S7

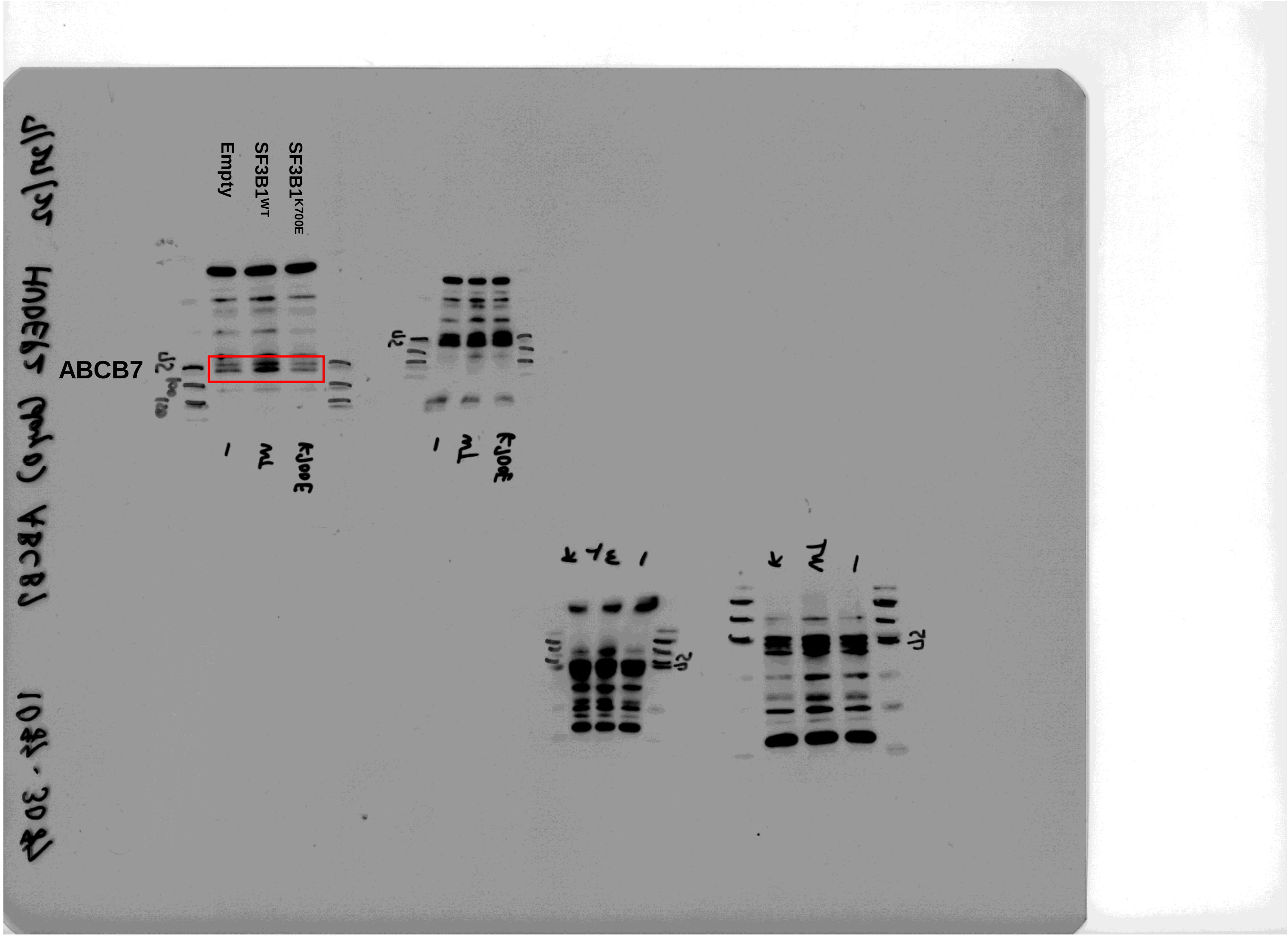

Supplementary Figure S7

Original blot image of ABCB7 in Fig. 2a.

Supplementary Figure S8

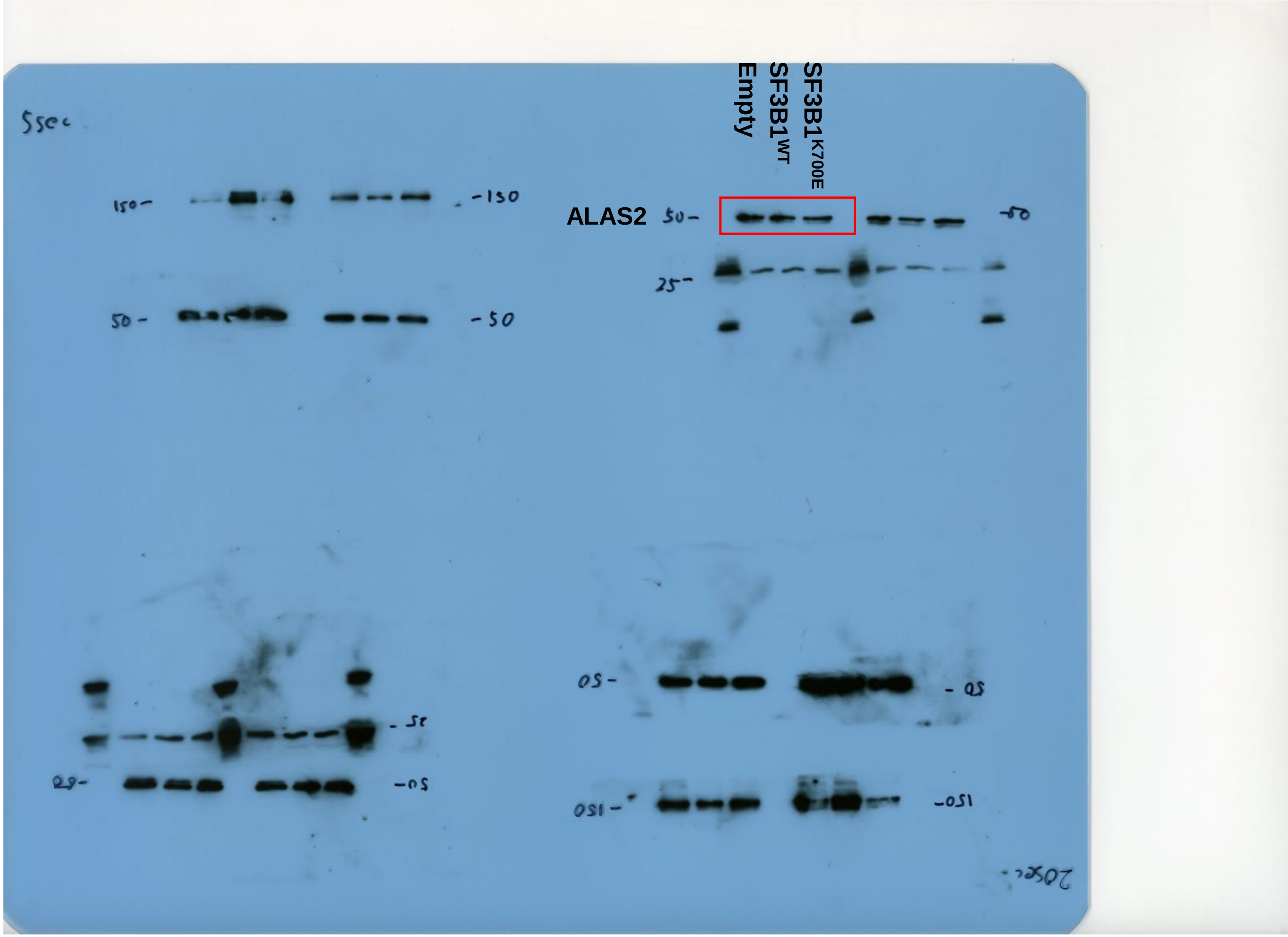

Supplementary Figure S8

Original blot image of ALAS2 in Fig. 2a.

Supplementary Figure S9

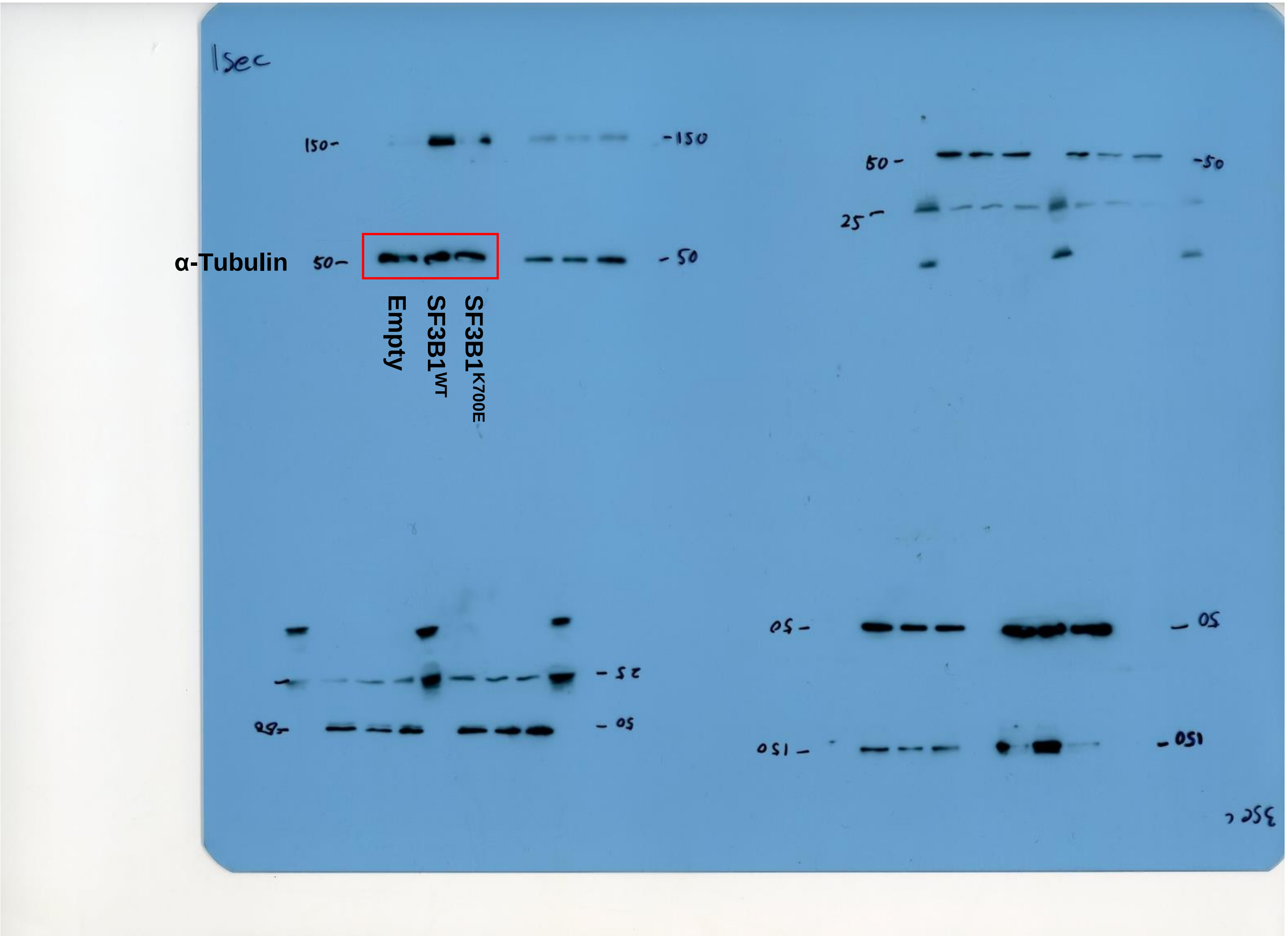

Supplementary Figure S9

Original blot image of α-Tubulin in Fig. 2a.

Supplementary Figure S10

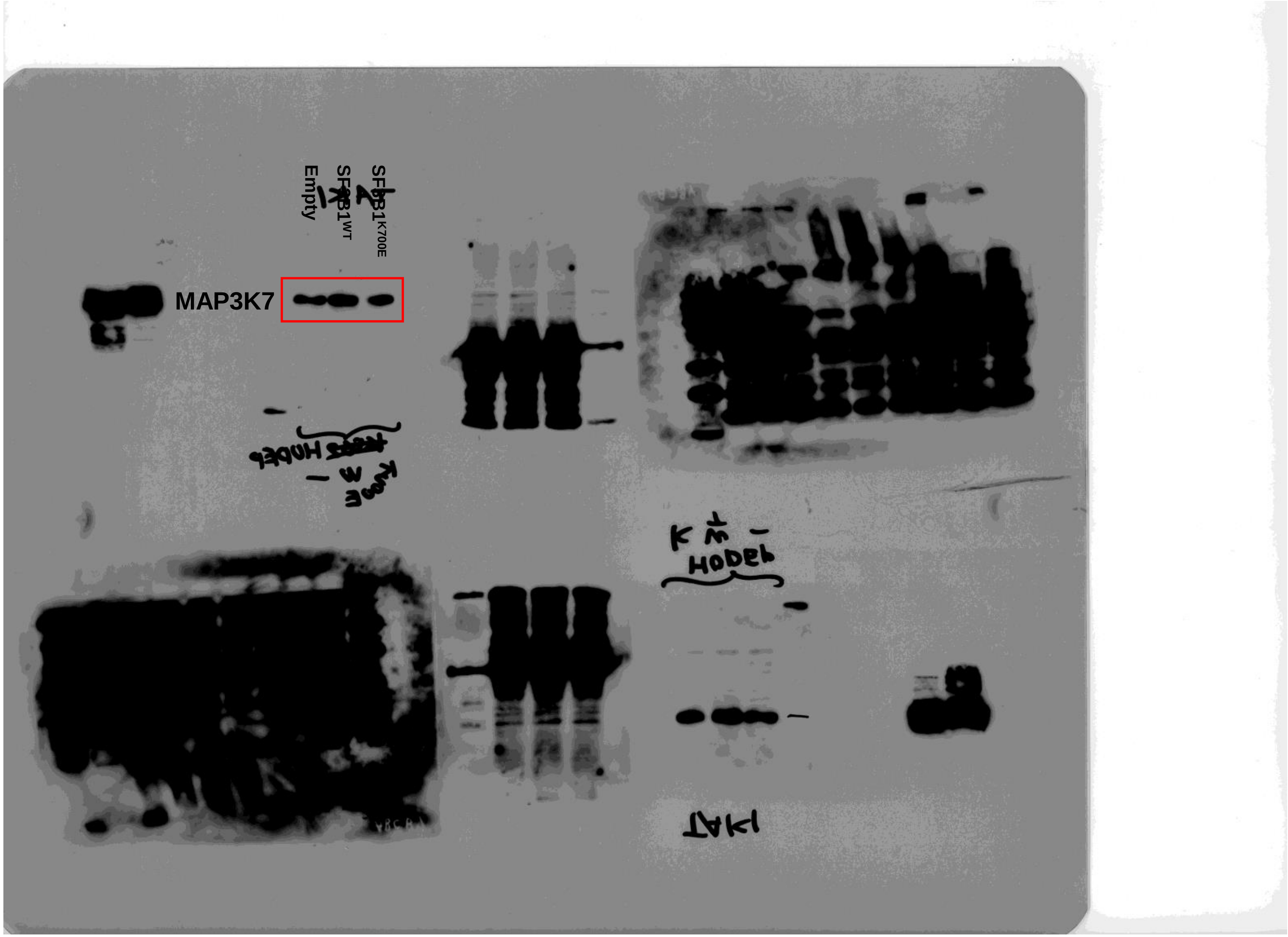

Supplementary Figure S10

Original blot image of MAP3K7 in Fig. 2c.

Supplementary Figure S11

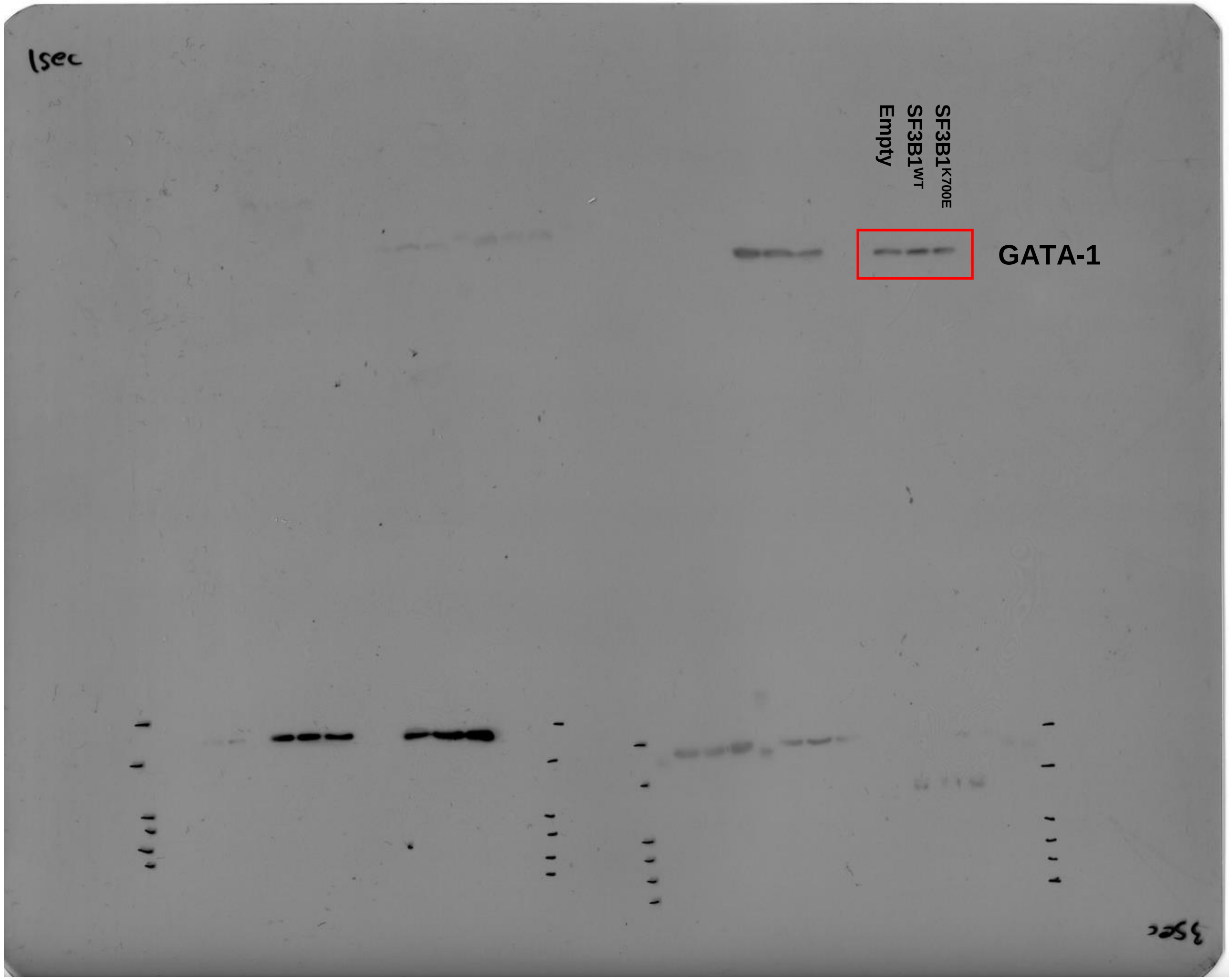

Supplementary Figure S11

Original blot image of GATA-1 in Fig. 2c.

Supplementary Figure S12

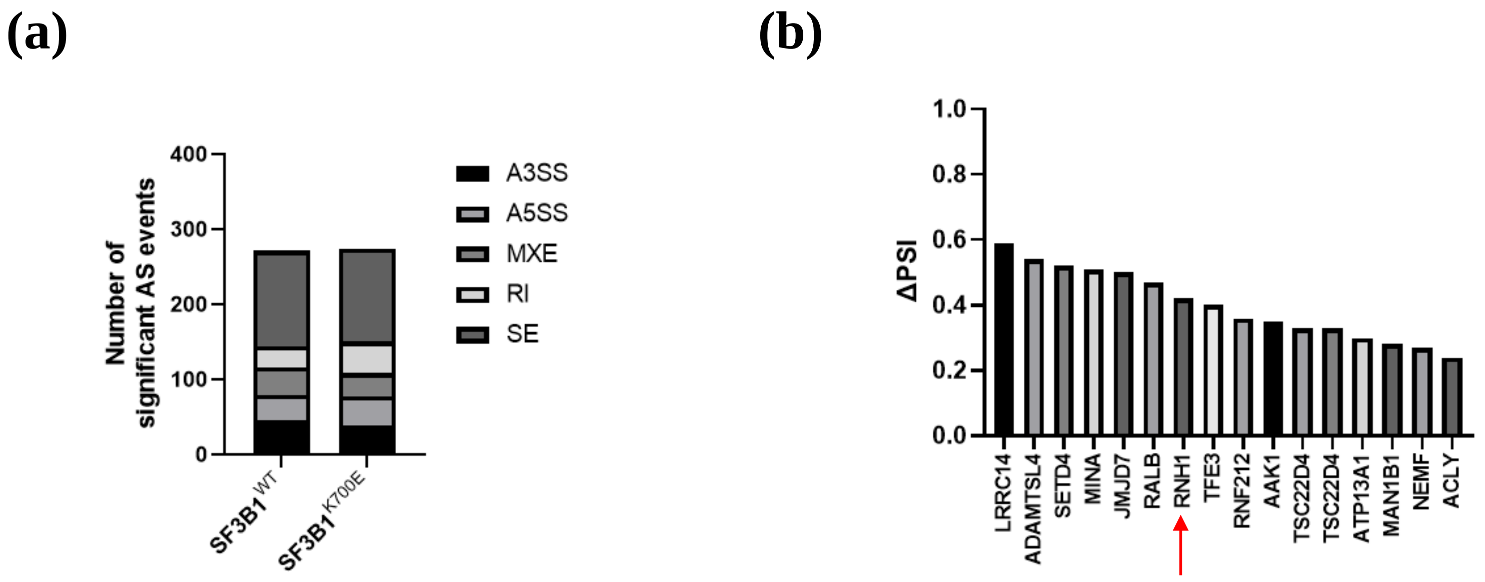

Supplementary Figure S12

Alternative splicing analysis for HUDEP-2 cells stably expressing SF3B1^{K700E}.

(a) Comprehensive AS analysis with MISO. The graph shows the number of significant AS events detected in HUDEP-2 cells stably expressing SF3B1^{WT} or SF3B1^{K700E} when compared with control vector-transduced HUDEP-2 cells.

(b) List of significant A3SS events showing increased PSI in HUDEP-2 cells expressing SF3B1^{K700E} in comparison to control vector-transduced HUDEP-2 cells.

Supplementary Figure S13

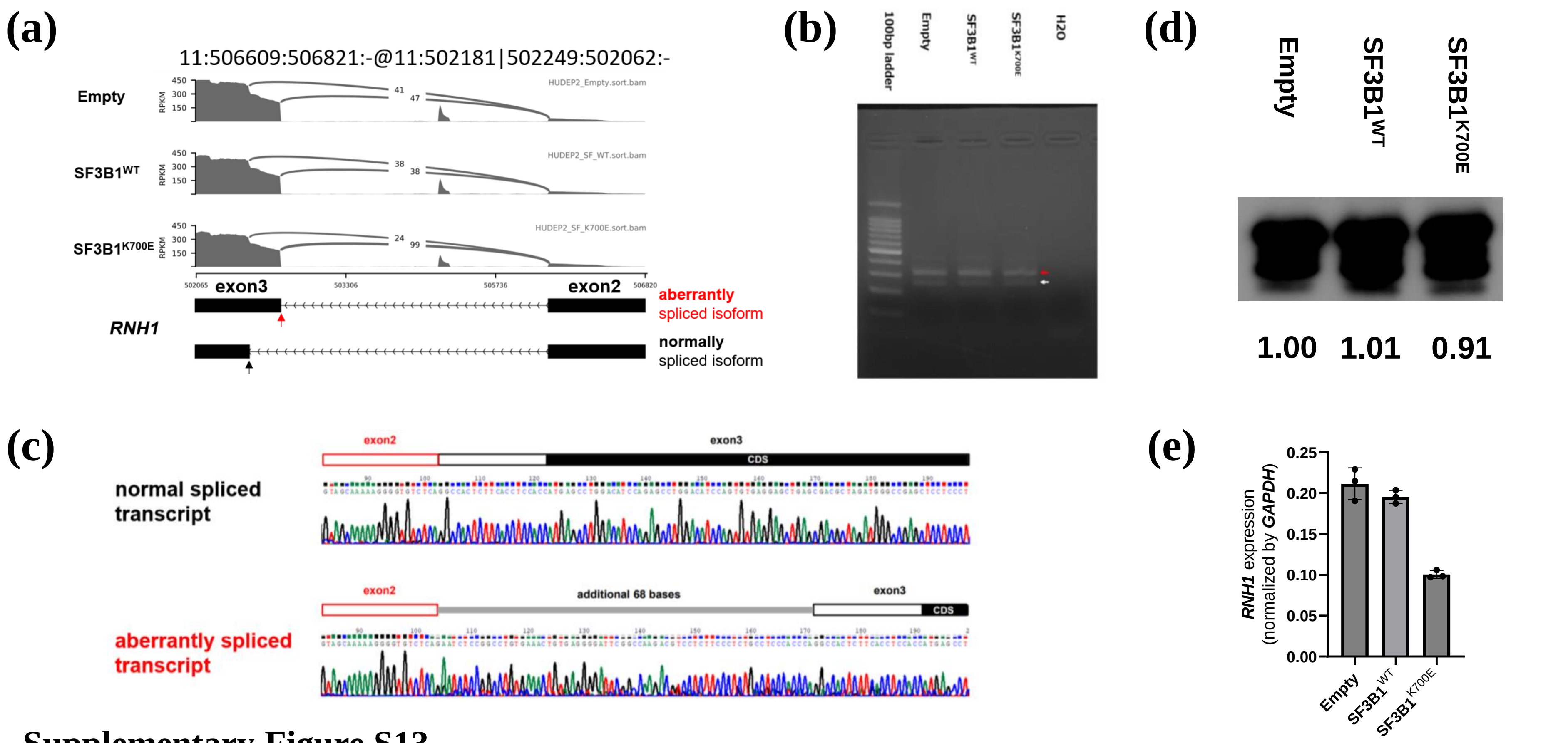

Supplementary Figure S13

Analysis for *RNH1* in HUDEP-2 cells stably expressing SF3B1^{K700E}.

(a) MISO sashimi plot of canonical and aberrant 3’ SS junctions between exons 2 and 3 of *RNH1* gene in HUDEP-2 cells expressing SF3B1^{K700E} or controls.

(b) Aberrant spliced isoforms of *RNH1* detected by RT-PCR. Red and white arrow indicate aberrantly and normally spliced isoform, respectively. The original gel image is presented in **Supplementary Figure S15**. **(c)** Sanger sequence data of normally and aberrantly spliced isoform of *RNH1*.

(d) Western blot analysis for RNH1. Relative expression levels of each gene in HUDEP-2 cells stably expressing SF3B1^{WT} or SF3B1^{K700E} in comparison to control vector-transduced HUDEP-2 cells are described under each picture.

(e) Expression levels of RNH1 were measured by quantitative RT-PCR (results shown as mean ± SD and dot plots).

Supplementary Figure S14

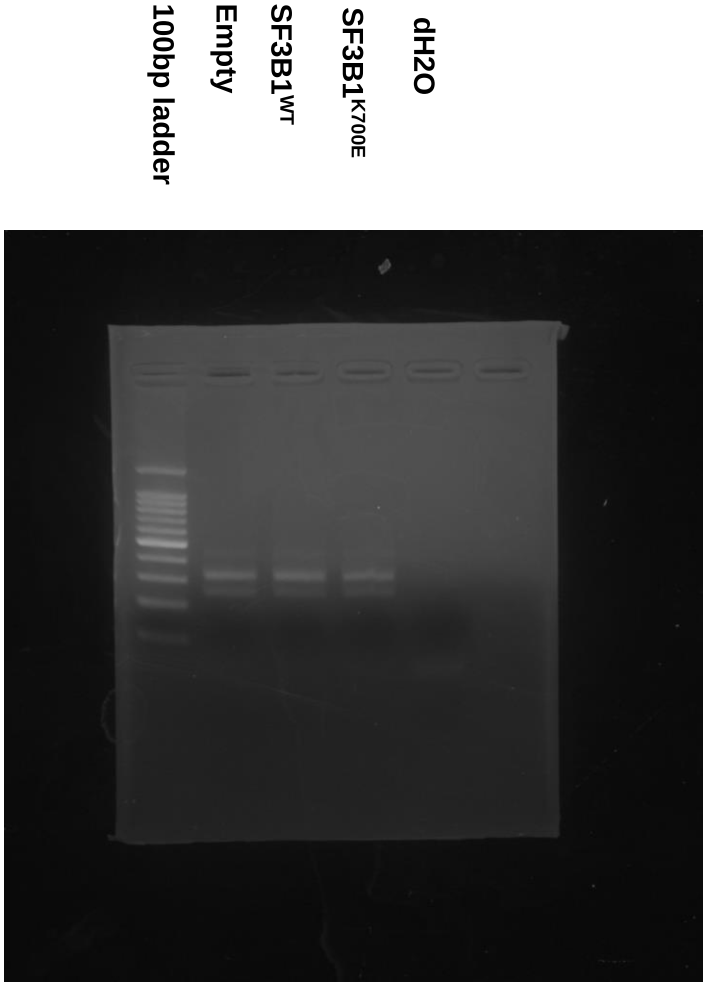

Supplementary Figure S14

Original gel image of Supplementary Fig. 13b.

Supplementary Figure S15

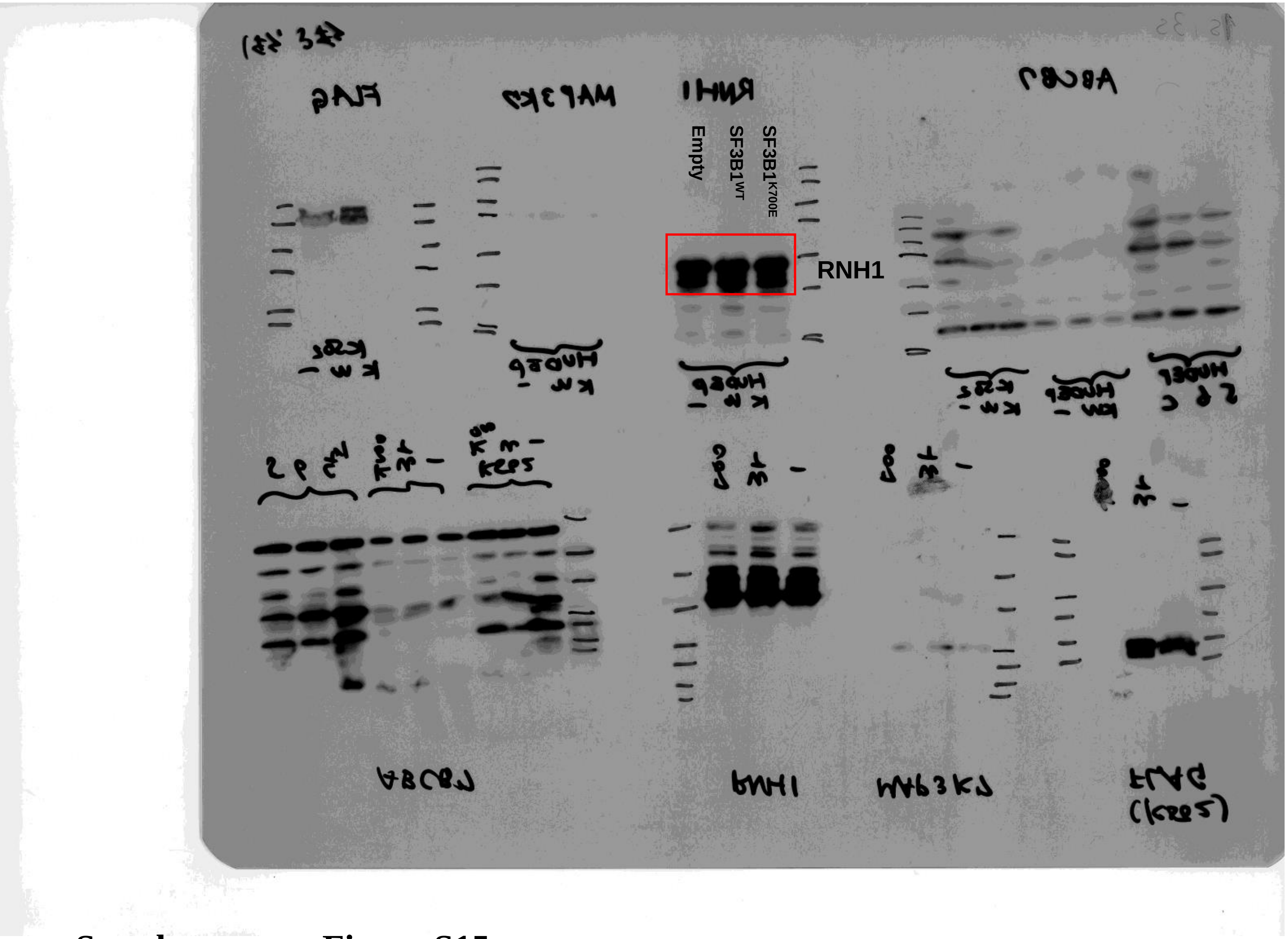

Supplementary Figure S15

Original blot image of RNH1 in Supplementary Fig. S13c.

Supplementary Figure S16

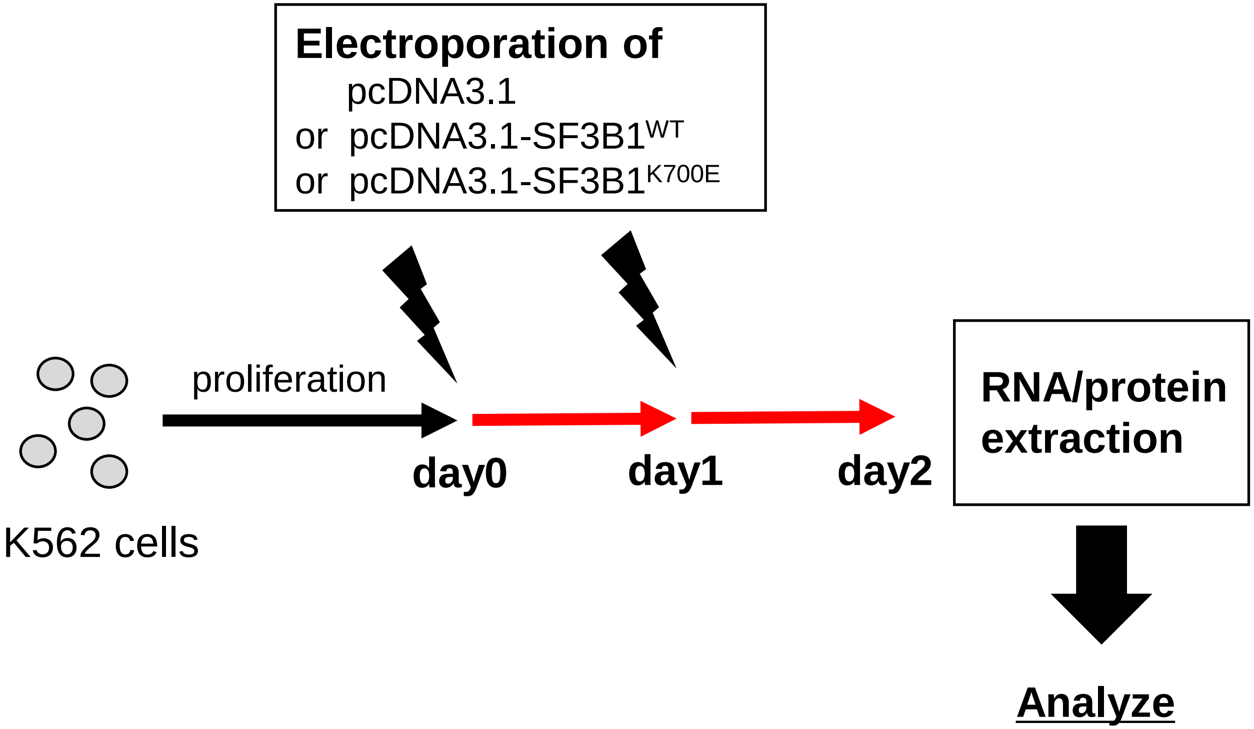

Supplementary Figure S16

Schematic outline of the procedures for transient overexpression of SF3B1^{K700E} in K562 cells.

pcDNA3.1 or pcDNA3.1-SF3B1^{WT} or pcDNA3.1-SF3B1^{K700E} was electroporated to K562 cells at day0 and day1. Extracted RNA and protein samples from each cell at day2 were analyzed.

Supplementary Figure S17

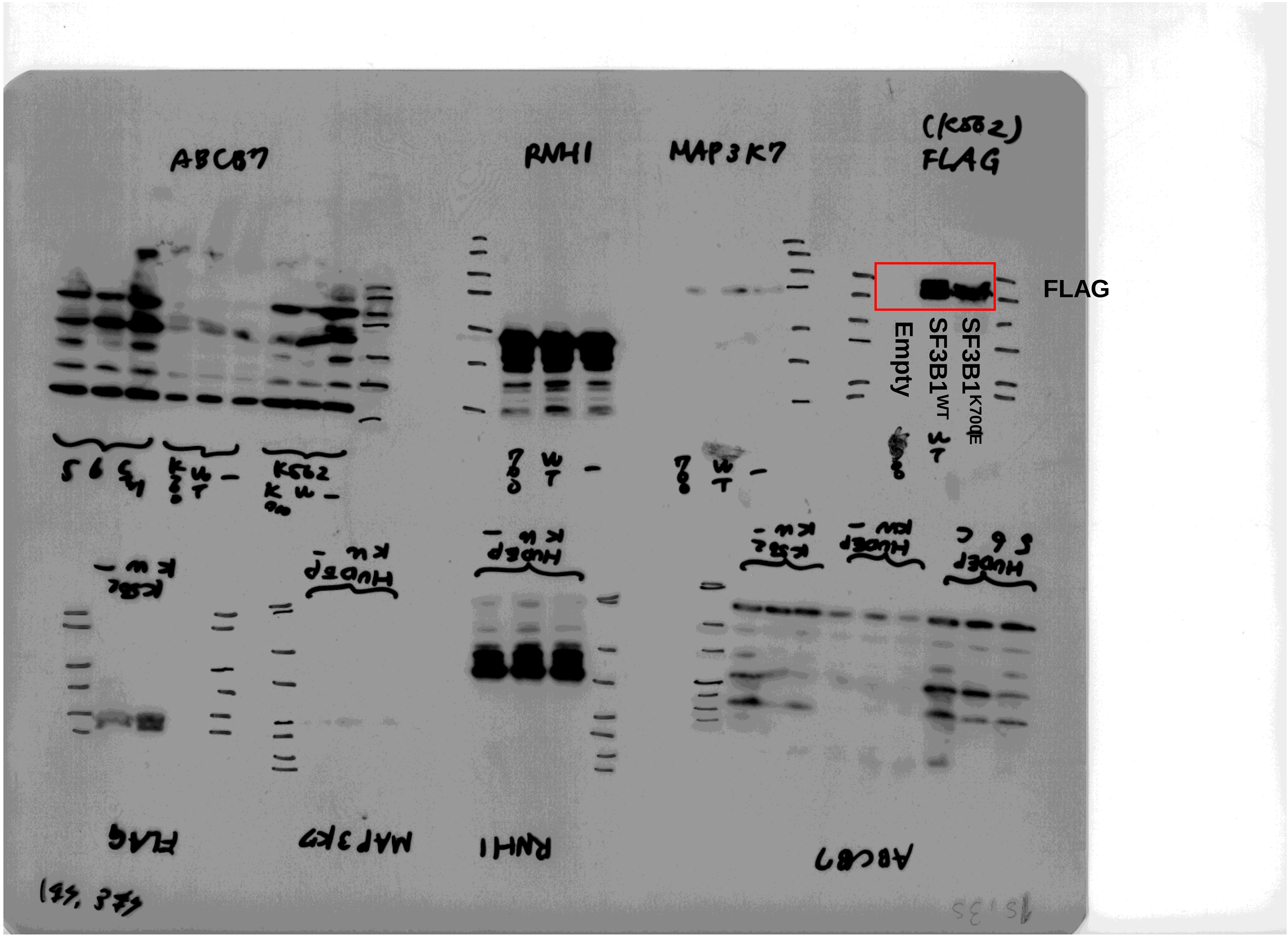

Supplementary Figure S17

Original blot image of FLAG in Fig. 4a.

Supplementary Figure S18

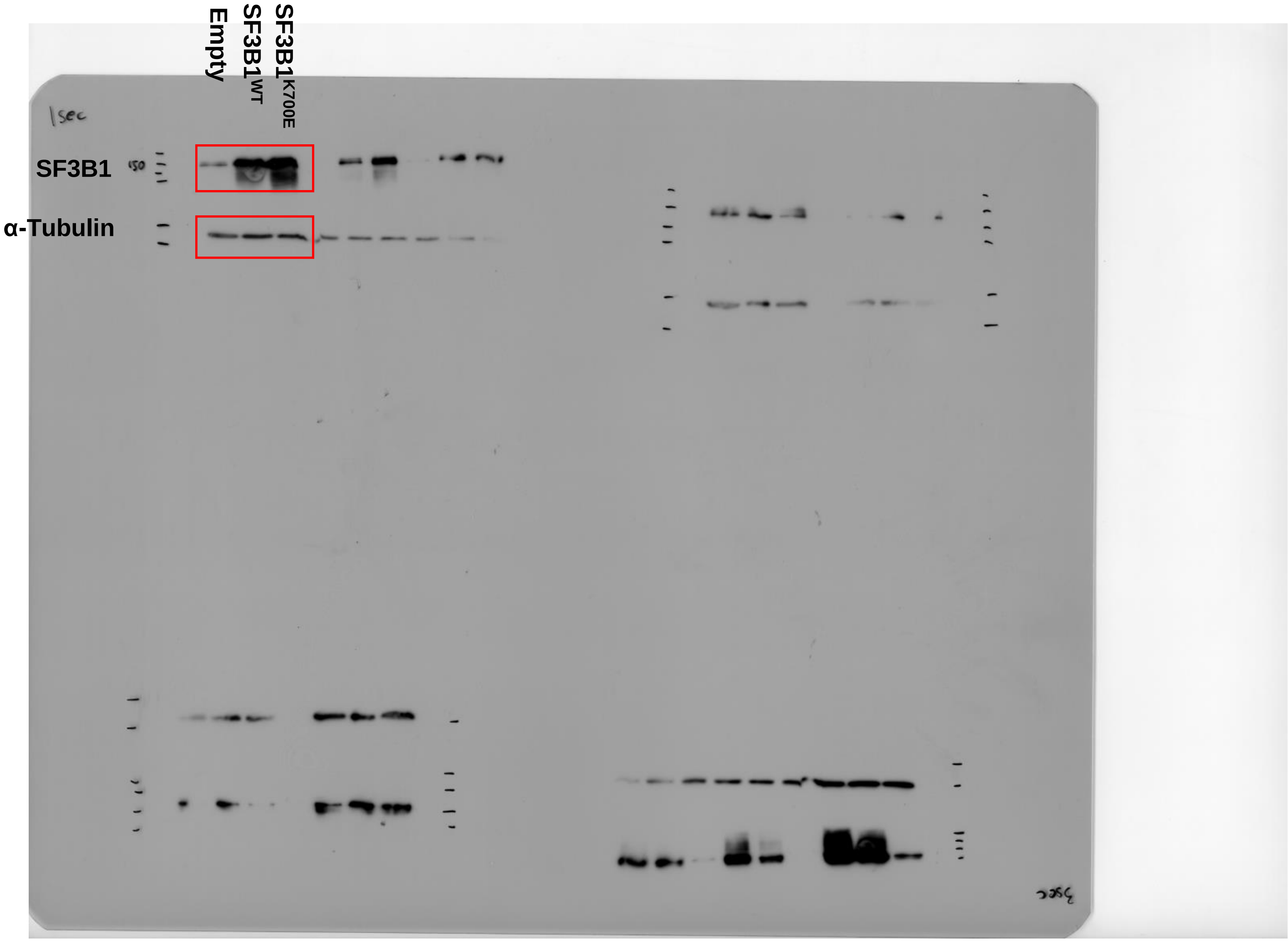

Supplementary Figure S18

Original blot image of SF3B1 and α-Tubulin in Fig. 4a.

Supplementary Figure S19

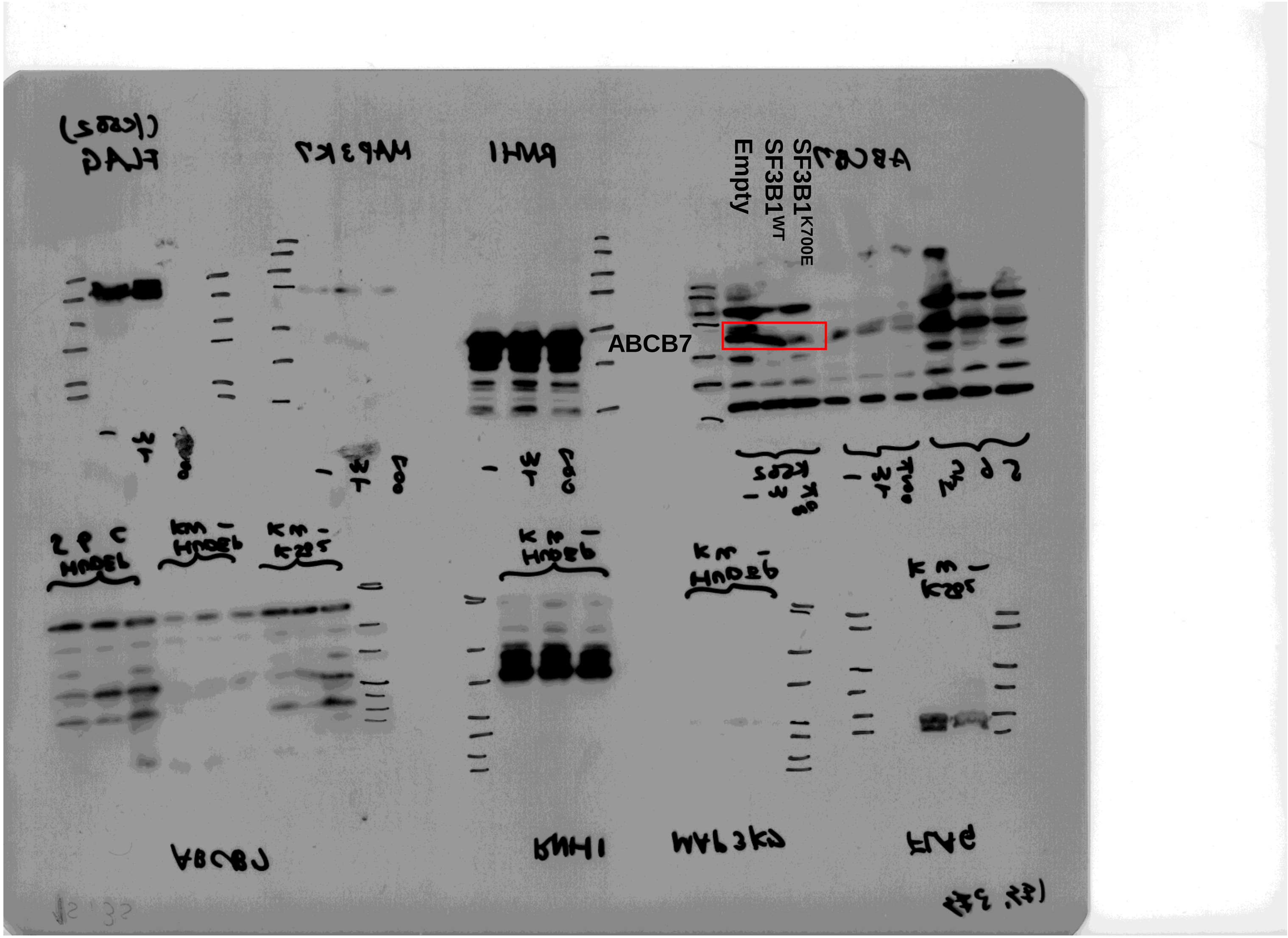

Supplementary Figure S19

Original blot image of ABCB7 in Fig. 4a.

Supplementary Figure S20

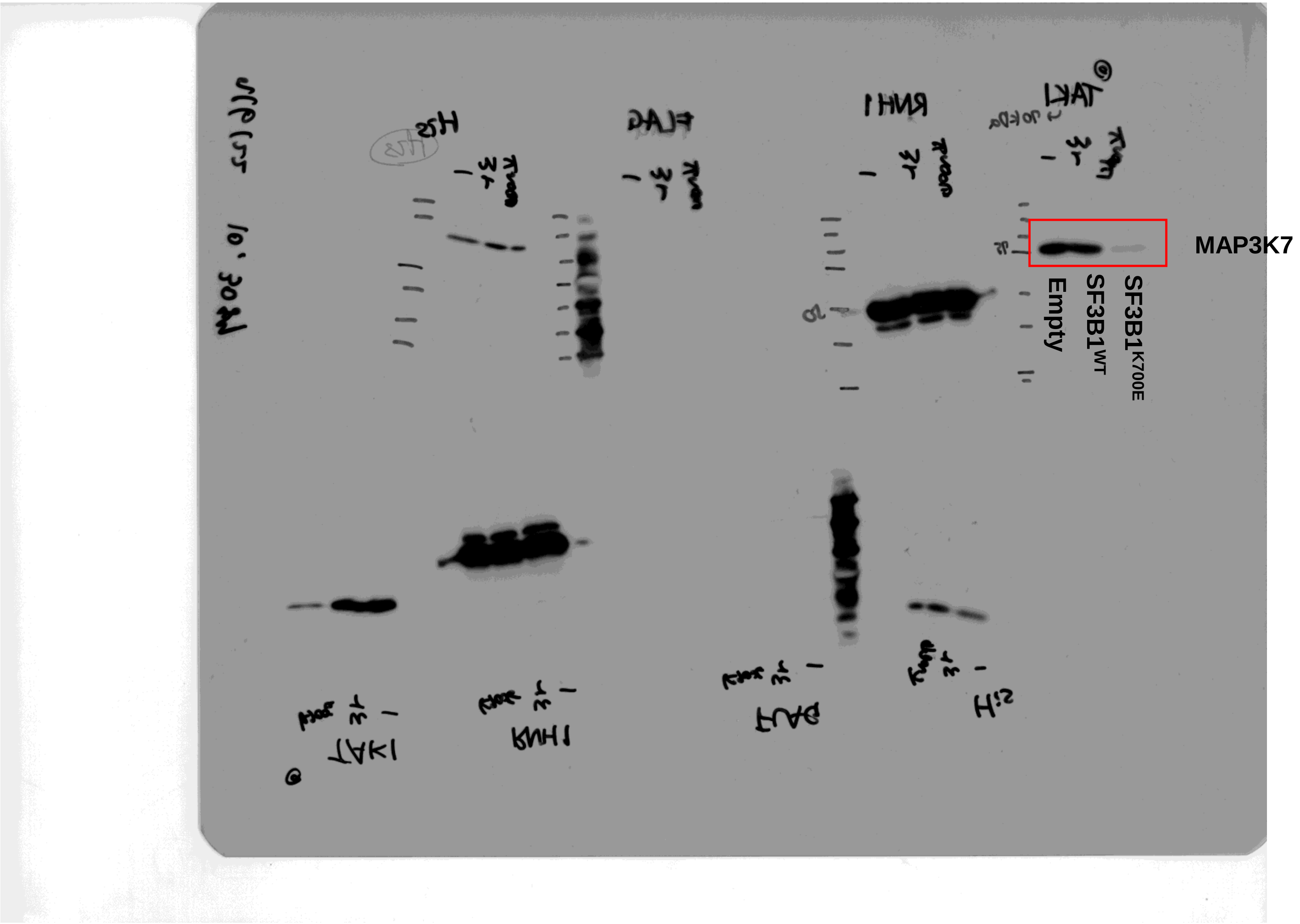

Supplementary Figure S20

Original blot image of MAP3K7 in Fig. 4a.

Supplementary Figure S21

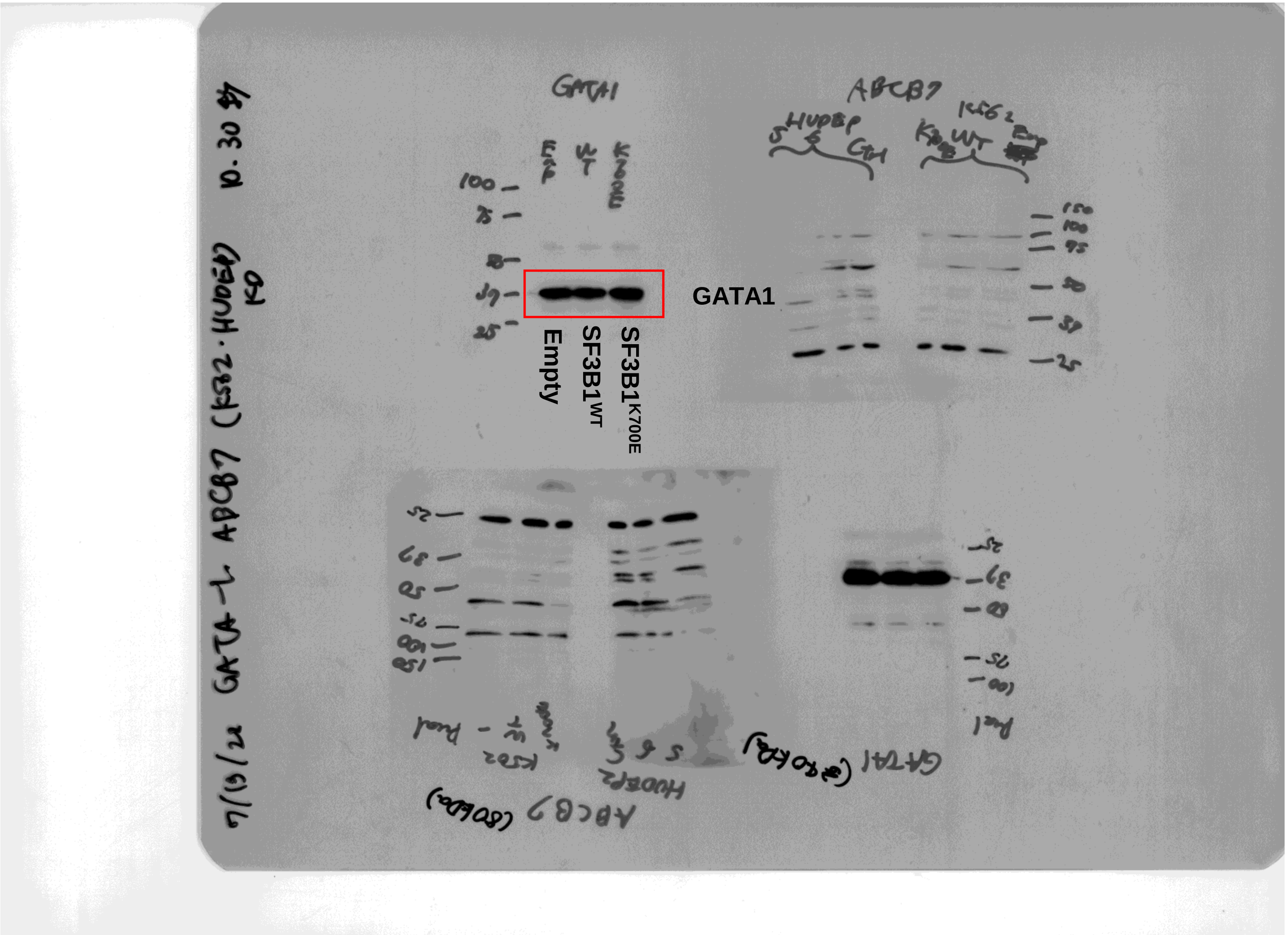

Supplementary Figure S21

Original blot image of GATA1 in Fig. 4a.

Supplementary Figure S22

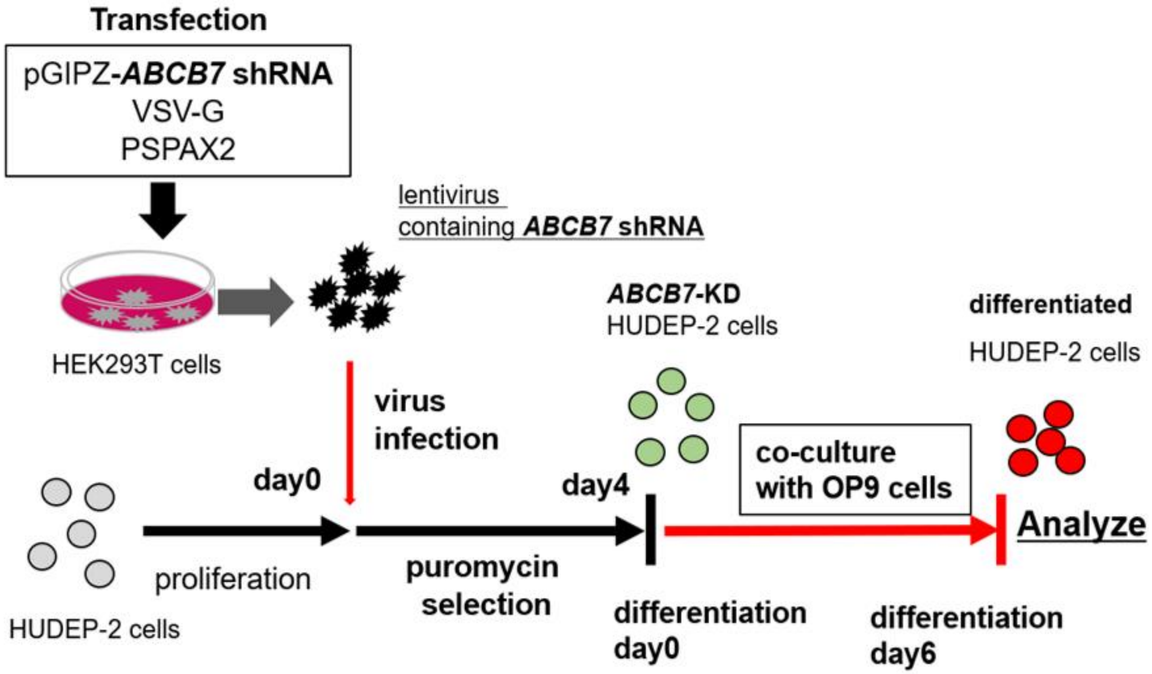

Supplementary Figure S22

Schematic outline of establishment of *ABCB7*-knockdown HUDEP-2 cells, followed by differentiation induction.

HUDEP-2 cells transduced with control shRNA or *ABCB7*-shRNA by lentivirus infection were purified with puromycin selection, and then were provided for co-culture with OP-9 cells for differentiation induction.

Supplementary Figure S23

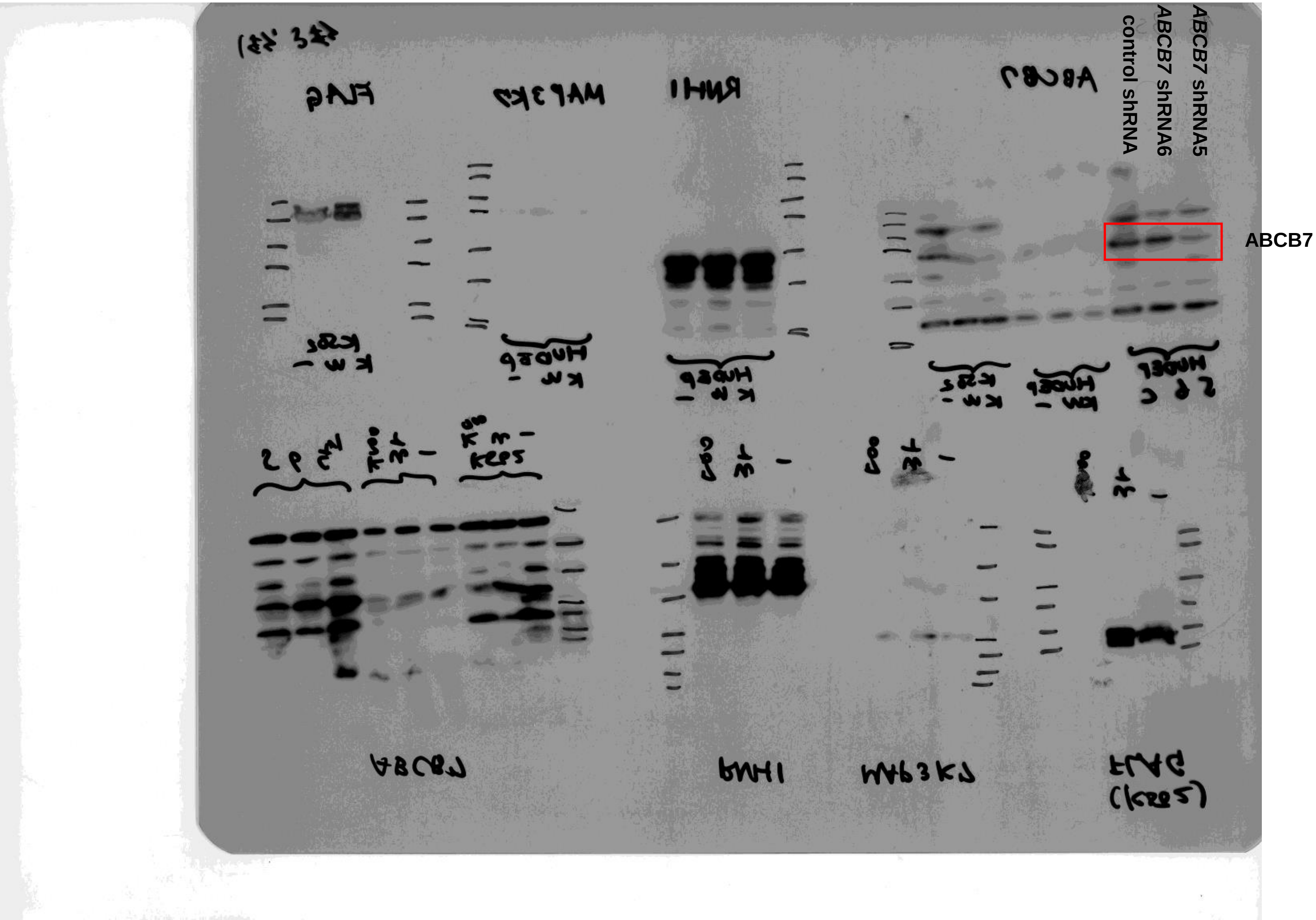

Supplementary Figure S23

Original blot image of ABCB7 in Fig. 5c.

Supplementary Figure S24

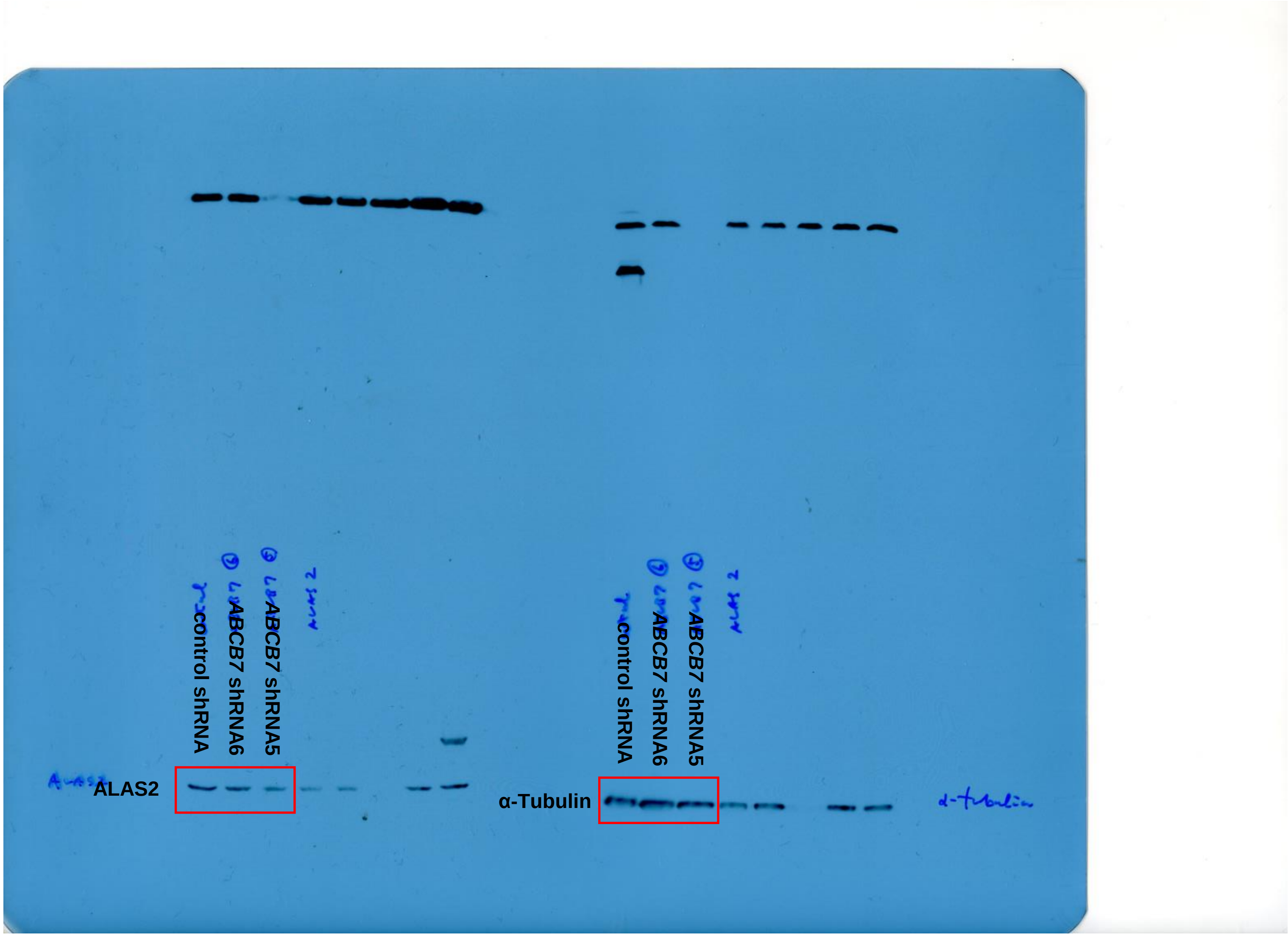

Supplementary Figure S24

Original blot image of ALAS2 and α-Tubulin in Fig. 5c.

Supplementary Figure S25

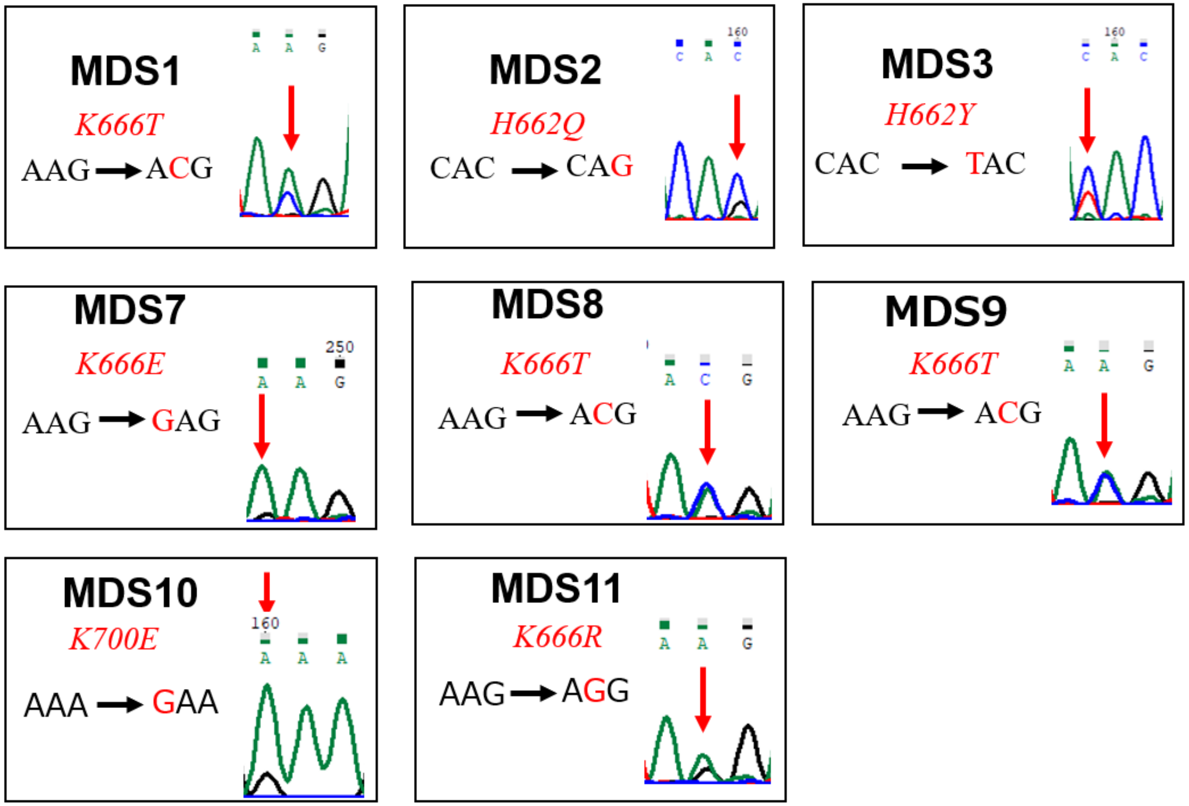

Supplementary Figure S25

Detection of *SF3B1* mutation among *SF3B1*^{MUT}-MDS patients diagnosed at Tohoku University Hospital.

Sanger sequence data indicating heterozygous *SF3B1* mutation in *SF3B1*^{MUT}-MDS patients are shown.

Supplementary Figure S26

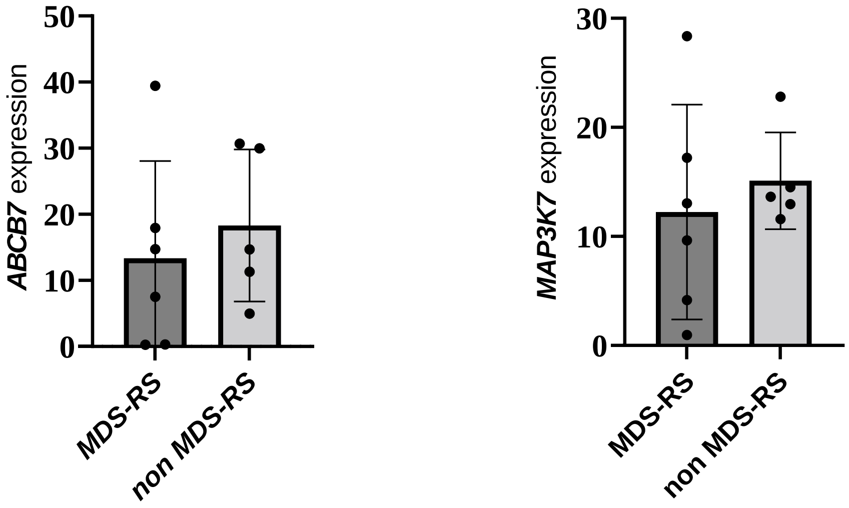

Supplementary Figure S26

Gene expression analysis for MDS-RS and non MDS-RS patients diagnosed at Tohoku University Hospital

We compared the expression levels of *ABCB7* and *MAP3K7* between MDS-RS (**n=7**) and non MDS-RS (**n=4**) patients diagnosed at Tohoku University Hospital.

Supplementary Figure S27

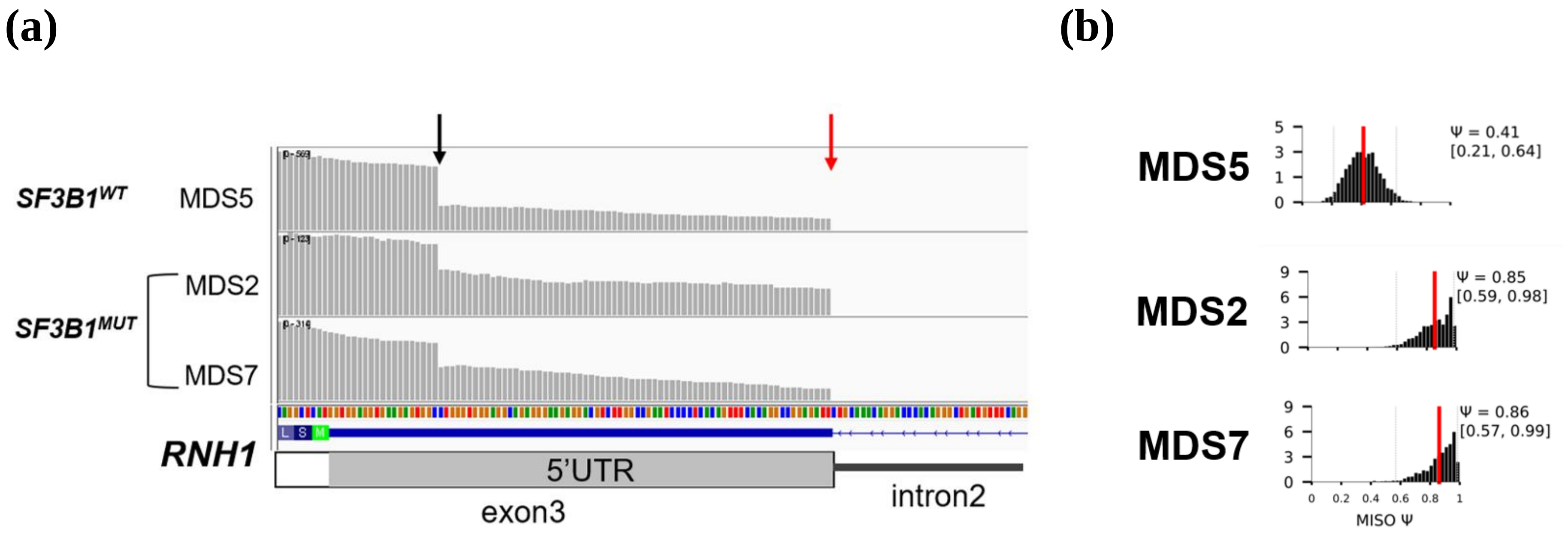

Supplementary Figure S27

Detection of alternative splicing in *RNH1* among MDS patients diagnosed at Tohoku University Hospital.

(a) Read coverage visualized by IGV around canonical 3′ SS of *RNH1* exon 3.
Black and red arrow indicate canonical and aberrant 3′ SS, respectively.

(b) Graphs indicating the distribution of MISO Ψ (which means incidence rate of a specific AS event calculated by MISO) for A3SS events in *RNH1* (11:506609:506821:-@11:502181|502249:502062:-). Mean MISO Ψ is shown by red vertical lines and 95% confidence intervals of MISO Ψ is shown by dotted grey vertical lines.

Supplementary Figure S28

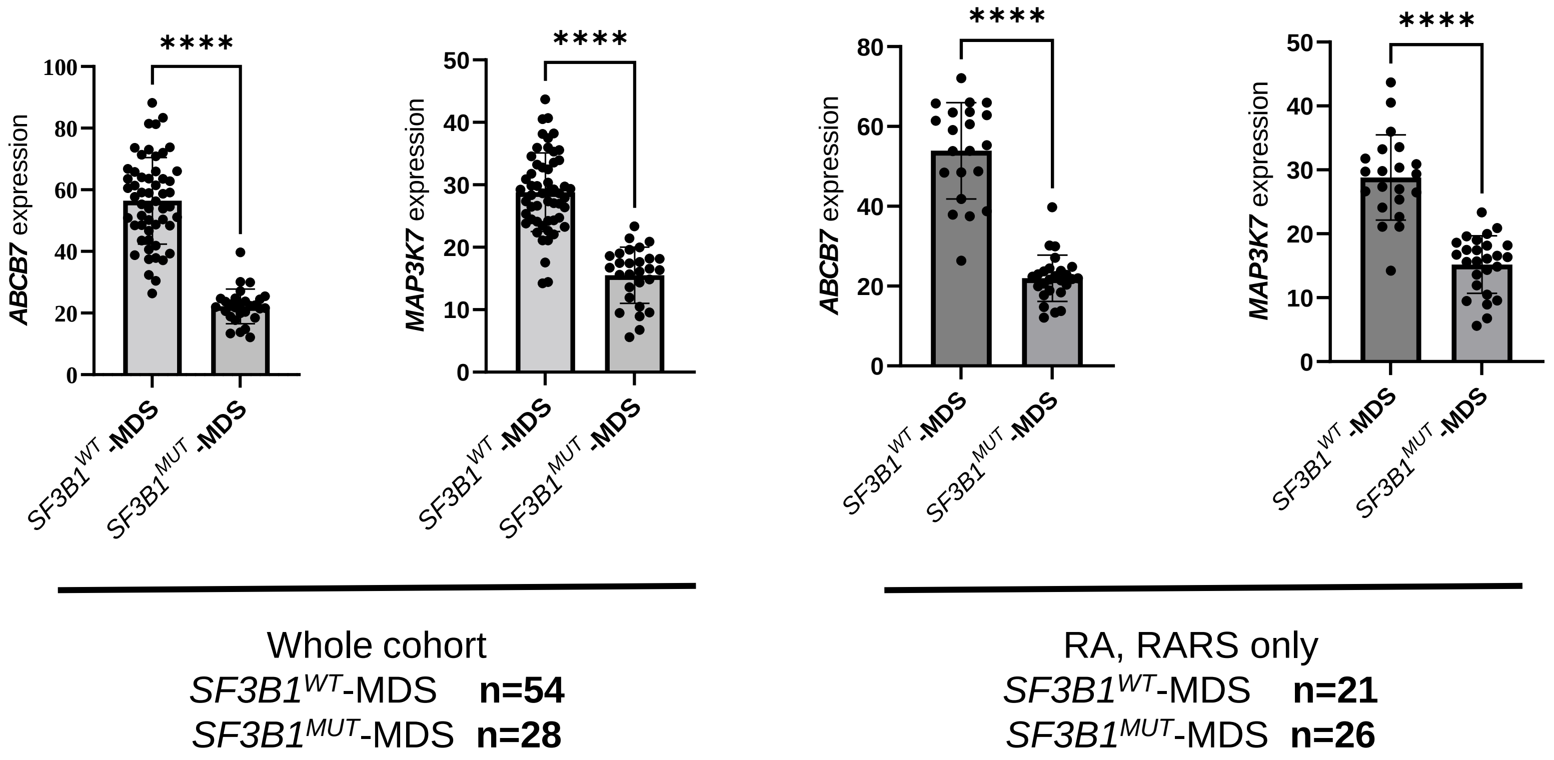

Supplementary Figure S28

Gene expression analysis for *SF3B1^{WT}*- and *SF3B1^{MUT}*-MDS based on GSE114922 dataset.

We compared the expression levels of *ABCB7*, *MAP3K7* of *SF3B1^{WT}*- and *SF3B1^{MUT}*-MDS patients included in the GSE114922 RNA-seq dataset.[35]

Supplementary Figure S29

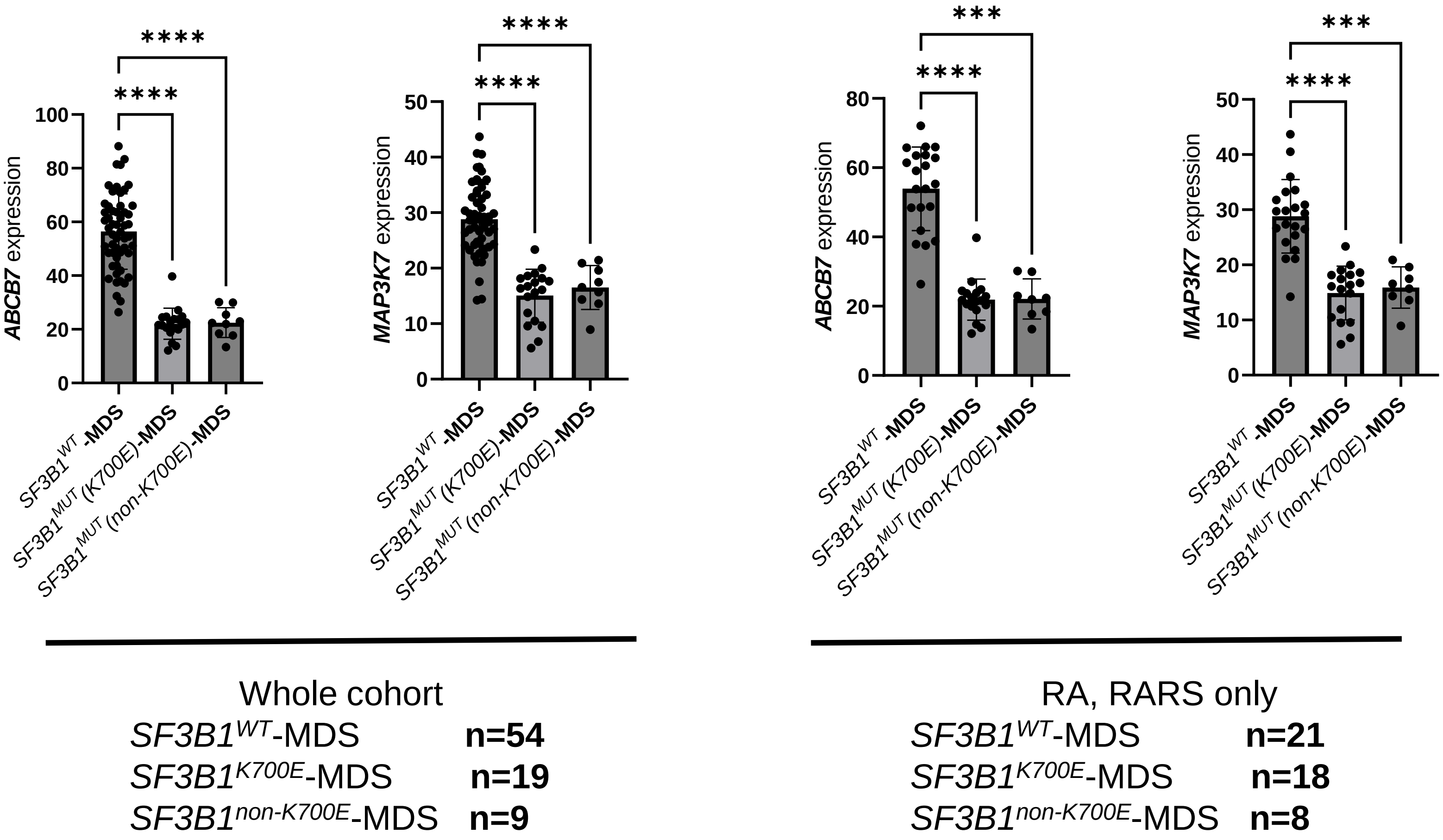

Supplementary Figure S29

Gene expression analysis for *SF3B1^WT*-, *SF3B1^K700E*- and SF3B1^non-K700E-MDS based on GSE114922 dataset.

We compared the expression levels of *ABCB7*, *MAP3K7* of *SF3B1^WT*-, *SF3B1^K700E*- and *SF3B1^non-K700E*-MDS patients included in the GSE114922 RNA-seq dataset.[35]

Supplementary Figure S30

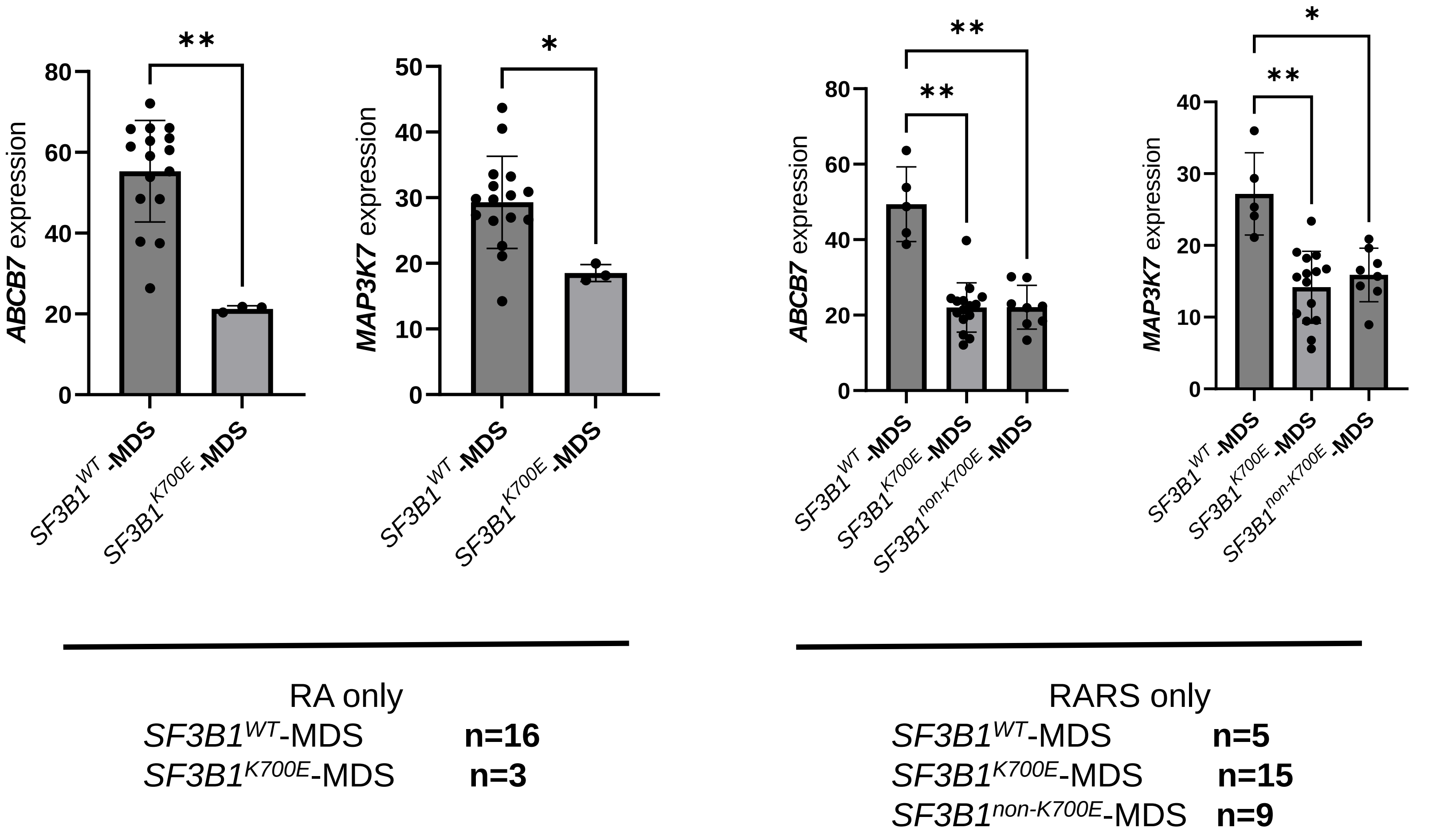

Supplementary Figure S30

Gene expression analysis for *SF3B1^{WT}*-, *SF3B1^{K700E}*- and SF3B1$^{non\text{-}K700E}$-MDS based on GSE114922 dataset.

We compared the expression levels of *ABCB7*, *MAP3K7* of *SF3B1^{WT}*-, *SF3B1^{K700E}*- and *SF3B1$^{non\text{-}K700E}$*-MDS patients included in the GSE114922 RNA-seq dataset.[35]

Supplementary Figure S31

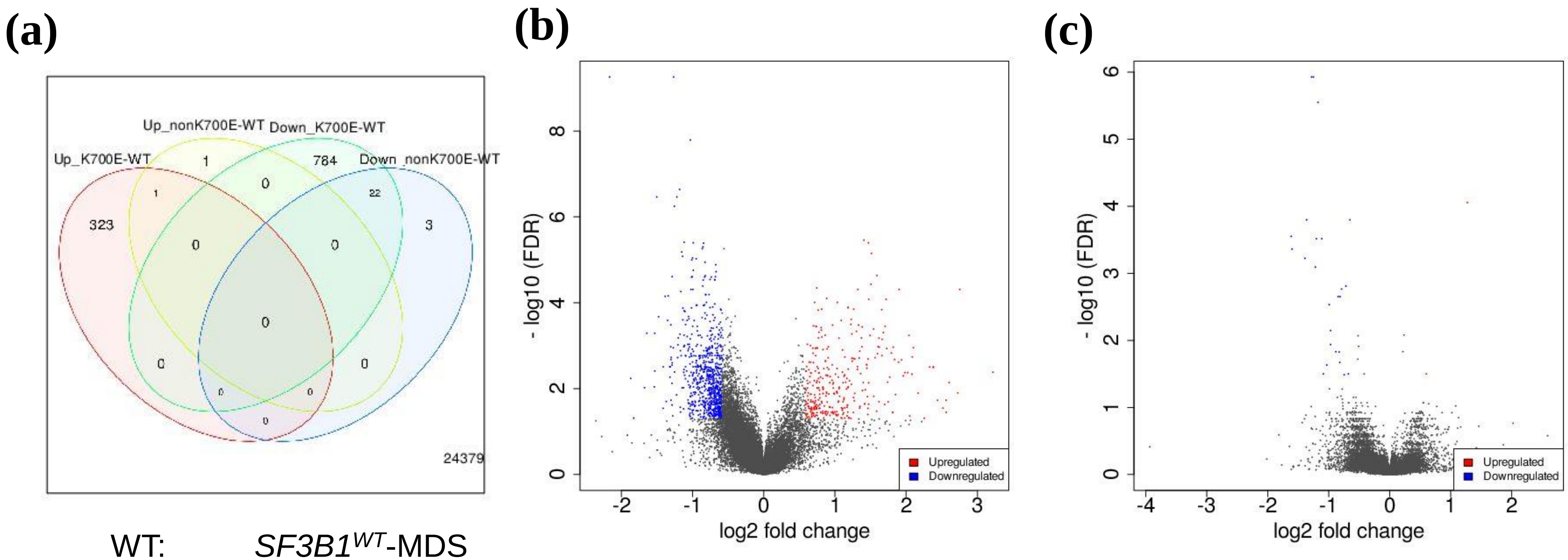

WT: *SF3B1*^{WT}-MDS
K700E: *SF3B1*^{K700E}-MDS
nonK700E: *SF3B1*$^{non\text{-}K700E}$-MDS

Supplementary Figure S31

Analysis of differentially expressed genes in MDS patients except CMML and RAEB according to SF3B1 mutation status and type of mutation using GSE114922 dataset.

(a) Venn diagram about the number of upregulated or downregulated genes in SF3B1^{K700E}- or SF3B1$^{non\text{-}K700E}$-MDS compared to SF3B1^{WT}-MDS.

(b) Volcano plot of differentially expressed genes in *SF3B1*^{K700E}-MDS compared to *SF3B1*^{WT}-MDS.

(c) Volcano plot of differentially expressed genes in *SF3B1*$^{non\text{-}K700E}$-MDS compared to *SF3B1*^{WT}-MDS.

Supplementary Figure S32

(a)

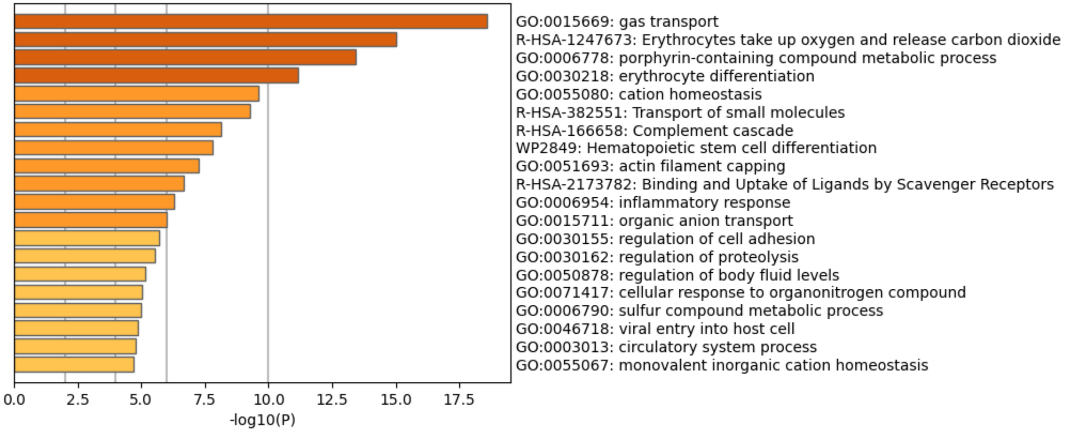

(b)

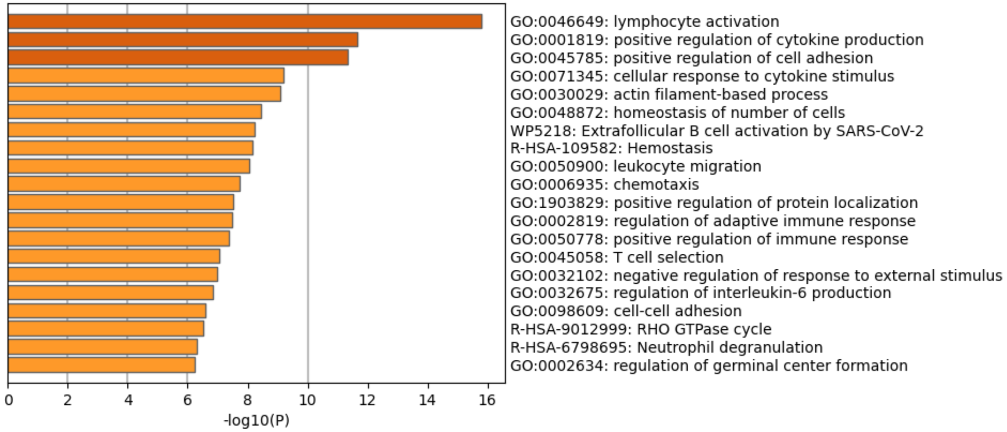

Supplementary Figure S32

GO enrichment analysis of dysregulated genes in $SF3B1^{K700E}$- compared to $SF3B1^{WT}$-MDS patients except CMML and RAEB using GSE114922 dataset.

Enrichment heatmap of genes (a) upregulated and (b) downregulated in $SF3B1^{K700E}$-MDS patients compared to $SF3B1^{WT}$-MDS patients.

Supplementary Figure S33

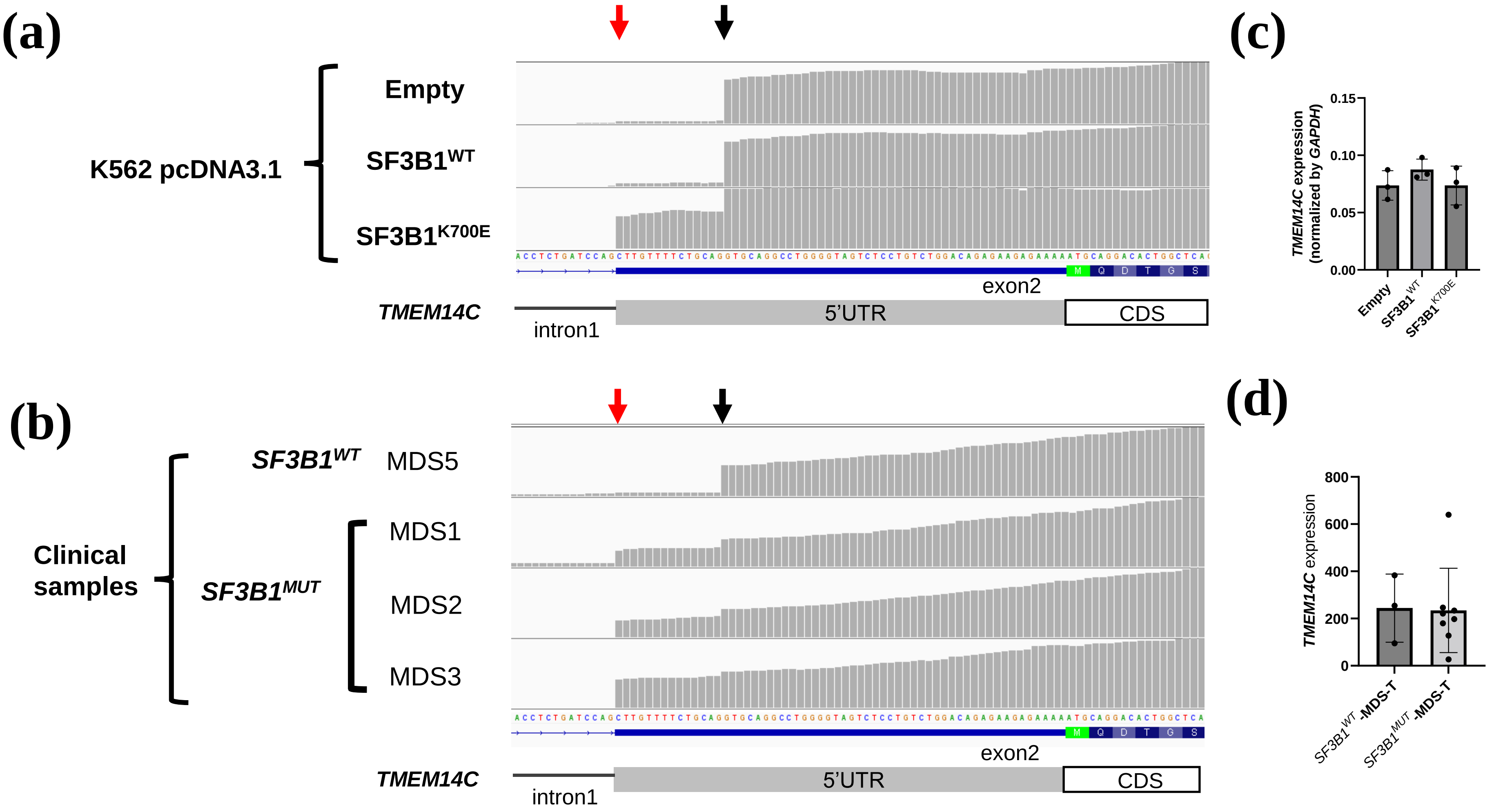

Supplementary Figure S33

Detection of A3SS events in TMEM14C in K562 overexpressing SF3B1^{K700E} and clinical samples.

(a), (b) Read-coverage visualized by IGV around canonical 3′ SS of TMEM14C exon 2 in K562 cells (a) and clinical samples (b). Black and red arrow indicate canonical and aberrant 3′ SS, respectively. (c) Expression levels of TMEM14C were measured by quantitative RT-PCR (results shown as mean ± SD and dot plots) in K562 cells. (d) RNA-seq analysis of TMEM14C expression levels in *SF3B1*^{WT}- or *SF3B1*^{MUT}-MDS patients diagnosed at Tohoku University Hospital.

SF3B1^{WT}- or *SF3B1*^{MUT}-MDS-T refers to the RNA-seq data of MDS patients diagnosed at Tohoku University Hospital.

Supplementary Figure S34

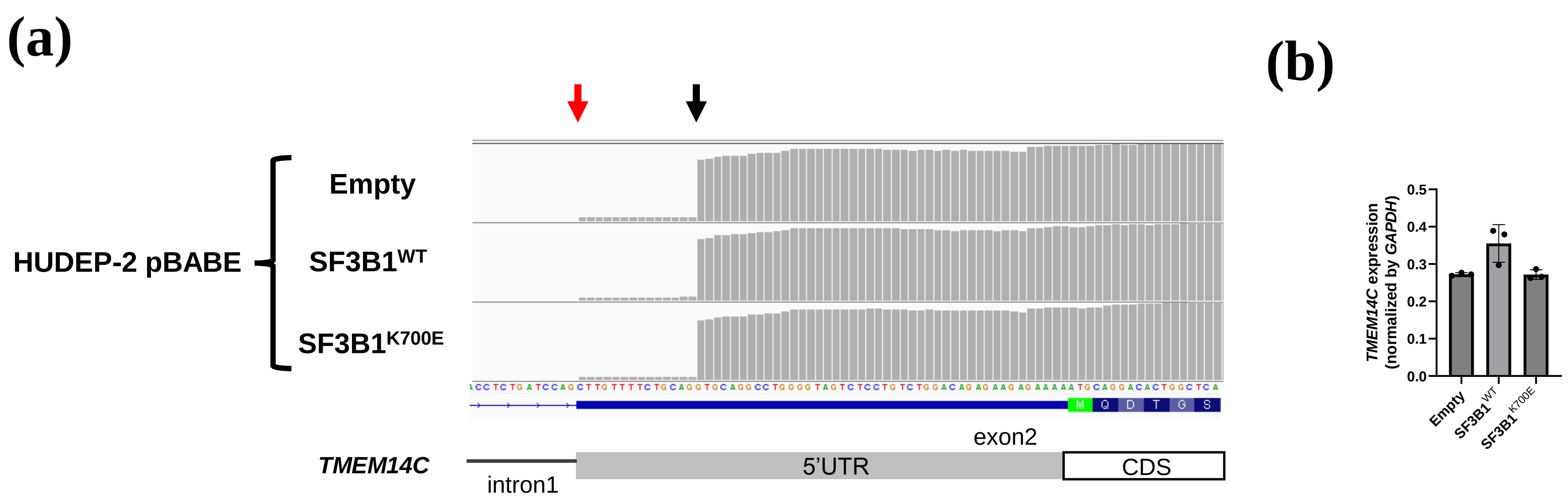

Supplementary Figure S34

Detection of A3SS events in TMEM14C in HUDEP2 stably expressing SF3B1^{K700E}.

(a) Read-coverage visualized by IGV around canonical 3′ SS of TMEM14C exon 2 in HUDEP-2 cells.
Black and red arrow indicate canonical and aberrant 3′ SS, respectively.
(b) Expression levels of TMEM14C were measured by quantitative RT-PCR (results shown as mean ± SD and dot plots) in HUDEP-2 cells.
